# Supplementary material for: Interplay between store-operated calcium entry and mitochondrial phosphate handling modulates force and fatigue during exercise
Source: bioRxiv. 2026 Jul 24:2025.05.22.655415. Preprint. [Version 3] doi: 10.1101/2025.05.22.655415 (PMC13420418; doi:10.1101/2025.05.22.655415)
Supplement: Supplementary file 1 [file NIHPP2025.05.22.655415v3-supplement-1.pdf]

bioRxiv preprint doi: https://doi.org/10.1101/2025.05.22.655415; this version posted July 24, 2026. The copyright holder for this preprint (which was not certified by peer review) is the author/funder, who has granted bioRxiv a license to display the preprint in perpetuity. It is made available under a CC-BY 4.0 International license.

Appendix A: Full model description

In our model, four major compartments (extracellular space, myoplasm, mitochondria, and SR lumen) are each divided into two well-mixed regions - one "junctional" region and one "bulk" region. Each junctional region is adjacent to the SR-T-tubule junction (either T-tubule volume, terminal myoplasm, junctional mitochondria, or terminal SR) and is treated as a separate compartment due to the highly localized ionic fluxes in the region. The myoplasm and extracellular space are separated by the plasma membrane (consisting of T-tubules and sarcolemma), whereas the myoplasm and SR lumen are separated by the SR membrane. In contrast, the junctional and bulk regions of each major compartment other than mitochondria are not separated by membranes. Accordingly, we assume that fluxes across the associated interface (denoted by dashed lines in Figure 1) are purely diffusive. That is, for a given species c:

$$\frac{dc_{\text{bulk}}}{dt} = J_{c,\text{bulk}} + D_c \frac{A_{\text{bulk-junc}}(c_{\text{junc}} - c_{\text{bulk}})}{vol_{\text{bulk}} \Delta d_{\text{diff}}}, \tag{A1}$$

$$\frac{dc_{\text{junc}}}{dt} = J_{c,\text{junc}} + D_c \frac{A_{\text{bulk-junc}}(c_{\text{bulk}} - c_{\text{junc}})}{vol_{\text{junc}} \Delta d_{\text{diff}}}, \tag{A2}$$

where Δd_{diff} is the characteristic length for diffusion, vol_{bulk} and vol_{junc} are the volumes of the bulk and junctional regions, respectively, and $A_{\text{bulk-junc}}$ is the area of the interface between the bulk and junctional regions. $J_{\text{c,bulk}}$ and $J_{\text{c,junc}}$ are the sum of reaction rates and other nondiffusive fluxes of species c in the bulk and junctional spaces, respectively. The above relation only applies to species in a volumetric compartment (SR lumen, myoplasm, or T-tubule volume). Variables associated with the state of surface species (e.g., ion channels in the sarcolemma) are assumed to be non-diffusive, with the exception of membrane voltage, which diffuses rapidly between the sarcolemma and T-tubules (Equations (3) and (4)).

To calculate the volume and surface area of each compartment (sarcolemma, T-tubule, myoplasm, SR membrane, SR lumen, and mitochondrial volume), we approximate the muscle fiber as a cylinder of radius 20 μm, matching the size of mouse fast twitch fibers (Wallinga et al., 1999; Luff and Atwood, 1972). The total volume and sarcolemma surface area are given by the volume and surface area of a cylindrical cross-section. We assume the fiber is homogeneous along the longitudinal direction, such that the length of our cylindrical volume of interest is arbitrary. The subcellular volumes (myoplasm, mitochondria, and SR lumen) are then calculated from previously determined volume fractions (Senneff and Lowery, 2021; Rincón et al., 2021), whereas the T-tubule and SR surface area are given by the surface area to volume ratios measured in (Peachey, 1965) and (Mobley and Eisenberg, 1975). The junctional myoplasmic volume is defined as the region within Δd_{diff} of the T-tubule surface, and, similarly, the junctional SR volume is defined as the volume within Δd_{diff} of the junctional SR membrane. The interface between the junctional SR and T-tubule membrane is assumed to occupy about 50% of the T-tubule surface, in good agreement with the 67% occupancy measured in frog skeletal muscle (Mobley and Eisenberg, 1975). Finally, the surface area of the junction between T-tubules and the extracellular space ($SA_{\text{EC,bulk-junc}}$)is computed assuming the volume fraction of T-tubules is constant throughout the fiber. All geometric parameters are summarized in Table A1 and expressions for individual subvolumes and surface areas are given in Table A2.

bioRxiv preprint doi: https://doi.org/10.1101/2025.05.22.655415; this version posted July 24, 2026. The copyright holder for this preprint (which was not certified by peer review) is the author/funder, who has granted bioRxiv a license to display the preprint in perpetuity. It is made available under a CC-BY 4.0 International license.

Table A1: Geometric parameters

Name	Description	Value	Reference
R_{fiber}	Fiber radius	20 µm	(Wallinga et al., 1999)
L_{fiber}	fiber length	100 µm	(arbitrary)
ϕ_{myo}	Myoplasm volume fraction	0.947	(Shorten et al., 2007)
ϕ_{SR}	SR volume fraction	0.05	(Shorten et al., 2007)
ϕ_{TT}	T-tubule volume fraction	0.003	(Mathias et al., 1977)
θ_{TT}	T-tubule volume-to-surface-area ratio	0.01 µm	(Peachey, 1965)
θ_{SR}	SR volume-to-surface-area ratio	0.1 µm	(Mobley and Eisenberg, 1975)
$\phi_{\text{SRM,TT-SR}}$	area fraction of SRM at SR-TT junction	0.5	(Mobley and Eisenberg, 1975)
ϕ_{Mito}	Mitochondria volume fraction (per myoplasm)	0.05	(Chen et al., 2001)

Table A2: Equations for sub-compartment volumes and surface areas

Major compartment	Subregion	Equation
Extracellular	Total	$vol_{\text{EC,tot}} \to \infty$
	Junctional	$vol_{\text{TT}} = \phi_{\text{TT}} \pi R_{\text{fiber}}^2 L_{\text{fiber}}$
	Bulk	$vol_{\text{EC}} \to \infty$
	Bulk-junc interface	$SA_{\text{EC,bulk-junc}} = \phi_{\text{TT}} 2\pi R_{\text{fiber}} L_{\text{fiber}}$
Plasma membrane	Total	$SA_{\text{PM}} = SA_{\text{SL}} + SA_{\text{TT}}$
	Junctional	$SA_{\text{TT}} = \dfrac{\phi_{\text{TT}} \pi R_{\text{fiber}}^2 L_{\text{fiber}}}{\theta_{\text{TT}}}$
	Bulk	$SA_{\text{SL}} = 2\pi R_{\text{fiber}} L_{\text{fiber}}$
Myoplasm	Total	$vol_{\text{myo}} = \phi_{\text{myo}}(1 - \phi_{\text{Mito}}) \pi R_{\text{fiber}}^2 L_{\text{fiber}}$
	Junctional	$vol_{\text{myo,junc}} = SA_{\text{TT}} \Delta d_{\text{diff}} (1 - \phi_{\text{Mito}})$
	Bulk	$vol_{\text{myo,bulk}} = vol_{\text{myo}} - vol_{\text{myo,junc}}$
	Bulk-junc interface	$SA_{\text{myo,bulk-junc}} = SA_{\text{TT}}(1 - \phi_{\text{SRM,TT-SR}})$
SR membrane	Total	$SA_{SRM} = \dfrac{\phi_{\text{SR}} \pi R_{\text{fiber}}^2 L_{\text{fiber}}}{\theta_{\text{SR}}}$
	Junctional	$SA_{\text{SRM,junc}} = SA_{\text{TT}} \phi_{\text{SRM,TT-SR}}$
	Bulk	$SA_{\text{SRM,bulk}} = SA_{\text{SRM}} - SA_{\text{SRM,junc}}$
SR	Total	$vol_{\text{SR}} = \phi_{\text{SR}} \pi R_{\text{fiber}}^2 L_{\text{fiber}}$
	Junctional	$vol_{\text{SR,junc}} = SA_{\text{SR,junc}} \Delta d_{\text{diff}}$
	Bulk	$vol_{\text{SR,bulk}} = vol_{\text{SR}} - vol_{\text{SR,junc}}$
	Bulk-junc interface	$SA_{\text{SR,bulk-junc}} = SA_{\text{SRM,junc}}$
Mito matrix	Total	$vol_{\text{Mito}} = \phi_{\text{myo}} \phi_{\text{Mito}} \pi R_{\text{fiber}}^2 L_{\text{fiber}}$
	Junctional	$vol_{\text{Mito,junc}} = SA_{\text{TT}} \Delta d_{\text{diff}} \phi_{\text{Mito}}$
	Bulk	$vol_{\text{Mito,bulk}} = vol_{\text{Mito}} - vol_{\text{Mito,junc}}$
	Bulk-junc interface	0

bioRxiv preprint doi: https://doi.org/10.1101/2025.05.22.655415; this version posted July 24, 2026. The copyright holder for this preprint (which was not certified by peer review) is the author/funder, who has granted bioRxiv a license to display the preprint in perpetuity. It is made available under a CC-BY 4.0 International license.

Table A3: Ionic fluxes summarized by region. References provided motivate the distribution of channels between junctional and bulk regions; in absence of additional evidence, we assume equal distribution across surface compartments.

Flux	ions (stoich.*)	TT	SL	Junctional SRM	Bulk SRM	Reference
J_{Na}	Na^+ (+1)	0.1	1	0	0	(Cannon et al., 1993)
J_{NaK}	Na^+ (-3), K^+ (+2)	0.1	1	0	0	(Wallinga et al., 1999)
J_{NCX}	Na^+ (+3), Ca^{2+} (-1)	1	1	0	0	This study
J_{KIR}	K^+ (+1)	1	1	0	0	(Ashcroft et al., 1985)
J_{KDR}	K^+ (-1)	0.45	1	0	0	(Wallinga et al., 1999)
$J_{\mathrm{Cl^-}}$	Cl^- (+1)	0.1	1	0	0	(Wallinga et al., 1999)
$J_{\mathrm{Ca^{2+}\ leak,M}}$	Ca^{2+} (+1)	1	1	0	0	This study
J_{SOCE}	Ca^{2+} (+1)	1	0	0	0	(Protasi et al., 2021)
J_{PMCA}	Ca^{2+} (-1)	1	1	0	0	This study
J_{RyR}	Ca^{2+} (+1)	0	0	1	0	(Senneff and Lowery, 2021)
J_{SERCA}	Ca^{2+} (-1)	0	0	0.1	1	(Scheibe et al., 2008)
$J_{\mathrm{Ca^{2+}\ leak,SR}}$	Ca^{2+} (+1)	0	0	1	1	(Wallinga et al., 1999)

*Stoichiometric coefficient is positive for fluxes into the myoplasm and negative for fluxes out of the myoplasm.

Table A4: All state variables and their default values.

Name	Description	Default value	Normalization const
**Junctional variables**			
V_{TT}	TT membrane voltage	$-88\,\mathrm{mV}$	$100\,\mathrm{mV}$
$P_{\mathrm{O,Orai1}}$	open probability of Orai1	0.0122	1
$[Na^+]_{\mathrm{TT}}$	Na^+ in TT volume	$1.47\times10^5\,\mu\mathrm{M}$	$1.47\times10^5\,\mu\mathrm{M}$
$[Na^+]_{\mathrm{myo,junc}}$	Na^+ in junctional myoplasm	$1.47\times10^4\,\mu\mathrm{M}$	$1\times10^3\,\mu\mathrm{M}$
m_{TT}	Na^+ channel activation variable in TT	0.0128	1
h_{TT}	Na^+ channel inactivation variable in TT	0.8051	1
S_{TT}	Na^+ channel slow inactivation variable in TT	0.8487	1
$[K^+]_{\mathrm{TT}}$	K^+ in T-tubule volume	$4\times10^3\,\mu\mathrm{M}$	4000
$[K^+]_{\mathrm{myo,junc}}$	K^+ in junctional myoplasm	$1.545\times10^5\,\mu\mathrm{M}$	$1\times10^5\,\mu\mathrm{M}$
n_{TT}	KDR activation variable in TT	0.003	1
$h_{\mathrm{K,TT}}$	KDR inactivation variable in TT	0.9983	1
$[Cl^-]_{\mathrm{TT}}$	Cl^- in T-tubule volume	$1.28\times10^5\,\mu\mathrm{M}$	$1.28\times10^5\,\mu\mathrm{M}$
$[Cl^-]_{\mathrm{myo,junc}}$	Cl^- in junctional myoplasm	$5.83\times10^3\,\mu\mathrm{M}$	$1\times10^3\,\mu\mathrm{M}$
$[Ca^{2+}]_{\mathrm{TT}}$	Ca^{2+} in T-tubule volume	$1.3\times10^3\,\mu\mathrm{M}$	$1.3\times10^3\,\mu\mathrm{M}$
$[Ca^{2+}]_{\mathrm{myo,junc}}$	Ca^{2+} in junctional myoplasm	$0.1\,\mu\mathrm{M}$	$0.1\,\mu\mathrm{M}$
$[Ca^{2+}]_{\mathrm{SR,junc}}$	Ca^{2+} in junctional SR	$1.5\times10^3\,\mu\mathrm{M}$	$1.5\times10^3\,\mu\mathrm{M}$
w_{RyR}	slow inactivation variable for RyR	0.9091	1

Continued on next page

bioRxiv preprint doi: https://doi.org/10.1101/2025.05.22.655415; this version posted July 24, 2026. The copyright holder for this preprint (which was not certified by peer review) is the author/funder, who has granted bioRxiv a license to display the preprint in perpetuity. It is made available under a CC-BY 4.0 International license.

Table A4: (continued) State variables

Name	Description	Default value	Normalization const
$[Ca^{2+}\text{-Parv}]_{\text{myo,junc}}$	Ca^{2+}-Parv in junctional myoplasm	0 µM	500 µM
$[Mg^{2+}\text{-Parv}]_{\text{myo,junc}}$	Mg^{2+}-Parv in junctional myoplasm	0 µM	1×10^3 µM
$[\text{ATP}]_{\text{myo,junc}}$	Free ATP in junctional myoplasm	8×10^3 µM	1 µM
$[Ca^{2+}\text{-ATP}]_{\text{myo,junc}}$	Ca^{2+}-ATP in junctional myoplasm	0.3632 µM	1 µM
$[Mg^{2+}\text{-ATP}]_{\text{myo,junc}}$	Mg^{2+}-ATP in junctional myoplasm	0 µM	1 µM
$[\text{Pi}]_{\text{myo,junc}}$	Inorganic phosphate in junctional myoplasm	4×10^3 µM	1 µM
$[\text{Pi}]_{\text{SR,junc}}$	Inorganic phosphate in junctional SR	1.6×10^3 µM	1 µM
$[Ca^{2+}\text{-Pi}]_{\text{SR,junc}}$	Ca^{2+}-phosphate precipitate in junctional SR	0 µM	1 µM
$[\text{CSQN}]_{\text{SR,junc}}$	Free calsequestrin in junctional SR	1.5×10^4 µM	1×10^3 µM
$[\text{ADP}]_{\text{myo,junc}}$	ADP in the junctional myoplasm	100 µM	100 µM
$[Ca^{2+}]_{\text{Mito,junc}}$	Free Ca^{2+} in junctional mitochondria	0.1 µM	1 µM
$[\text{Pi}]_{\text{Mito,junc}}$	Phosphate in junctional mitochondria	5×10^3 µM	1×10^3 µM
$[Ca^{2+}\text{-Pi}]_{\text{Mito,junc}}$	Ca^{2+}-phosphate in junctional mitochondria	0 µM	100 µM
$[\text{ATP}]_{\text{Mito,junc}}$	ATP in junctional mitochondria	5×10^3 µM	1×10^3 µM
$[\text{NADH}]_{\text{Mito,junc}}$	NADH in junctional mitochondria	50 µM	100 µM
$V_{\text{Mito,junc}}$	Junctional mitochondrial membrane potential	150 mV	100 mV
**Bulk variables**			
V_{SL}	SL membrane voltage	−88 mV	100 mV
$[Na^+]_{\text{myo,bulk}}$	Na^+ in bulk myoplasm	1.47×10^4 µM	1×10^3 µM
m_{SL}	Na^+ channel activation variable in SL	0.0128	1
h_{SL}	Na^+ channel inactivation variable in SL	0.8051	1
S_{SL}	Na^+ channel slow inactivation variable in SL	0.8487	1
$[K^+]_{\text{myo,bulk}}$	K^+ in bulk myoplasm	1.545×10^5 µM	1×10^5 µM
n_{SL}	KDR activation variable in SL	0.003	1
$h_{\text{K,SL}}$	KDR inactivation variable in SL	0.9983	1
$[Cl^-]_{\text{myo,bulk}}$	Cl^- in bulk myoplasm	5.83×10^3 µM	1×10^3 µM
$[Ca^{2+}]_{\text{myo,bulk}}$	Ca^{2+} in bulk myoplasm	0.1 µM	0.1 µM
$[Ca^{2+}]_{\text{SR,bulk}}$	Ca^{2+} in bulk SR	1.5×10^3 µM	1.5×10^3 µM
$[Ca^{2+}\text{-Parv}]_{\text{myo,bulk}}$	Ca^{2+}-Parv in bulk myoplasm	0 µM	500 µM
$[Mg^{2+}\text{-Parv}]_{\text{myo,bulk}}$	Mg^{2+}-Parv in bulk myoplasm	0 µM	1×10^3 µM
$[\text{ATP}]_{\text{myo,bulk}}$	Free ATP in bulk myoplasm	8×10^3 µM	1 µM
$[Ca^{2+}\text{-ATP}]_{\text{myo,bulk}}$	Ca^{2+}-ATP in bulk myoplasm	0.3632 µM	1 µM
$[Mg^{2+}\text{-ATP}]_{\text{myo,bulk}}$	Mg^{2+}-ATP in bulk myoplasm	0 µM	1 µM

Continued on next page

bioRxiv preprint doi: https://doi.org/10.1101/2025.05.22.655415; this version posted July 24, 2026. The copyright holder for this preprint (which was not certified by peer review) is the author/funder, who has granted bioRxiv a license to display the preprint in perpetuity. It is made available under a CC-BY 4.0 International license.

Table A4: (continued) State variables

Name	Description	Default value	Normalization const
$[\text{Pi}]_{\text{myo,bulk}}$	Inorganic phosphate in bulk myoplasm	4×10^3 µM	1 µM
$[\text{Pi}]_{\text{SR,bulk}}$	Inorganic phosphate in bulk SR	1.6×10^3 µM	1 µM
$[\text{Ca}^{2+}\text{-Pi}]_{\text{SR,bulk}}$	Ca^{2+}-phosphate precipitate in bulk SR	0 µM	1 µM
$[\text{CSQN}]_{\text{SR,bulk}}$	Free calsequestrin in bulk SR	1.5×10^4 µM	1×10^3 µM
$[\text{Trop-Ca}^{2+}]$	Troponin-Ca^{2+} in bulk myoplasm	0 µM	1 µM
$[\text{Trop-2Ca}^{2+}]$	Troponin-$2Ca^{2+}$ in bulk myoplasm	0 µM	1 µM
$[D]$	Unblocked, detached cross-bridge density	0 µM	1 µM
$[A_{\text{pre}}]$	Attached cross-bridge density, pre-power stroke	0 µM	1 µM
$[A_{\text{post}}]$	Attached cross-bridge density, post-power stroke	0 µM	1 µM
$[\text{ADP}]_{\text{myo,bulk}}$	ADP in the bulk myoplasm	100 µM	100 µM
$[\text{Ca}^{2+}]_{\text{Mito,bulk}}$	Free Ca^{2+} in bulk mitochondria	0.1 µM	1 µM
$[\text{Pi}]_{\text{Mito,bulk}}$	Phosphate in bulk mitochondria	5×10^3 µM	1×10^3 µM
$[\text{Ca}^{2+}\text{-Pi}]_{\text{Mito,bulk}}$	Ca^{2+}-phosphate in bulk mitochondria	0 µM	100 µM
$[\text{ATP}]_{\text{Mito,bulk}}$	ATP in bulk mitochondria	5×10^3 µM	1×10^3 µM
$[\text{NADH}]_{\text{Mito,bulk}}$	NADH in bulk mitochondria	50 µM	100 µM
$V_{\text{Mito,bulk}}$	Bulk mitochondrial membrane potential	150 mV	100 mV

The equations used in our system of ordinary differential equations (ODEs) were adapted from different papers that incorporated Ca^{2+} signaling and action potential generation within mammalian muscle fibers. Reactions/fluxes are given for each module in Table A5. Throughout, whenever a given flux or variable is associated with both bulk and junctional regions, we denote the intracellular region with subscript i, the extracellular region with subscript o, and the associated region of the plasma membrane (either T-tubules or bulk sarcolemma) with subscript M. For instance, we write all Nernst potentials as:

$$E_{\text{Na}^+,\text{M}} = \frac{RT}{F}\ln\left(\frac{[\text{Na}^+]_\text{o}}{[\text{Na}^+]_\text{i}}\right) \tag{A3}$$

$$E_{\text{K}^+,\text{M}} = \frac{RT}{F}\ln\left(\frac{[\text{K}^+]_\text{o}}{[\text{K}^+]_\text{i}}\right) \tag{A4}$$

$$E_{\text{Cl}^-,\text{M}} = -\frac{RT}{F}\ln\left(\frac{[\text{Cl}^-]_\text{o}}{[\text{Cl}^-]_\text{i}}\right) \tag{A5}$$

$$E_{\text{Ca}^{2+},\text{M}} = \frac{RT}{2F}\ln\left(\frac{[\text{Ca}^{2+}]_\text{o}}{[\text{Ca}^{2+}]_\text{i}}\right), \tag{A6}$$

where [M,i,o] can either be [‘TT’,‘myo,junc’,‘TT volume’] or [‘SL’,‘myo,bulk’,‘EC’]. Similarly, ‘SR’ and ‘Mito’ are used to refer to the associated junctional compartment (‘SR,junc‘, Mito,junc‘) or bulk compartment (‘SR,bulk’, ‘Mito,junc’) where applicable. The resulting ODEs are summarized in Table A6 and all associated parameters provided in Table A7.

bioRxiv preprint doi: https://doi.org/10.1101/2025.05.22.655415; this version posted July 24, 2026. The copyright holder for this preprint (which was not certified by peer review) is the author/funder, who has granted bioRxiv a license to display the preprint in perpetuity. It is made available under a CC-BY 4.0 International license.

Table A5: Reactions and fluxes

Name	Description	Equation
**Module 1**		
$I_{\mathrm{KIR,M}}$	inward rectifier current	$G_{\mathrm{K}}Q_{\mathrm{KIR}}\left(\frac{K_{\mathrm{R}}^2}{K_{\mathrm{K}}+K_{\mathrm{R}}^2}\right)\left(1-\left(1+\frac{K_{\mathrm{S}}\left(1+\frac{K_{\mathrm{R}}^2}{K_{\mathrm{K}}}\right)}{S_{\mathrm{K}}^2\exp\left(\frac{2(1-\delta)V_{\mathrm{M}}F}{RT}\right)}\right)^{-1}\right)(E_{\mathrm{K^+,M}}-V_{\mathrm{M}})$, where $K_{\mathrm{R}}=[\mathrm{K^+}]_{\mathrm{o}}\exp\left(-\frac{\delta E_{\mathrm{K^+,M}}F}{RT}\right)$
$I_{\mathrm{KDR,M}}$	delayed rectifier current	$g_{\mathrm{K}}Q_{\mathrm{KDR}}n_{\mathrm{M}}^4h_{\mathrm{K,M}}(E_{\mathrm{K^+,M}}-V_{\mathrm{M}})$
$I_{\mathrm{NaK,M}}$	$\mathrm{Na^+}$-$\mathrm{K^+}$ pump current	$\frac{Ff_{\mathrm{NaK}}Q_{\mathrm{NaK}}J_{\mathrm{NaK}}}{\left(1+\frac{K_{\mathrm{mK}}}{[\mathrm{K^+}]_{\mathrm{o}}}\right)^2\left(1+\frac{K_{\mathrm{mNa}}}{[\mathrm{Na^+}]_{\mathrm{i}}}\right)^3}\left(\frac{[\mathrm{ATP}]_{\mathrm{i}}}{k_{\mathrm{ATP}}+[\mathrm{ATP}]_{\mathrm{i}}}\right)$, where $f_{\mathrm{NaK}}=\left(1+0.12\exp\left(-\frac{V_{\mathrm{M}}F}{10RT}\right)+0.04\sigma_{\mathrm{M}}\exp\left(-\frac{V_{\mathrm{M}}F}{RT}\right)\right)^{-1}$ and $\sigma_{\mathrm{M}}=\frac{1}{7}\left(\exp\left(\frac{[\mathrm{Na^+}]_{\mathrm{o}}}{67300\ \mu\mathrm{M}}\right)-1\right)$
$I_{\mathrm{Na^+,M}}$	$\mathrm{Na^+}$ channel current	$g_{\mathrm{Na}}Q_{\mathrm{Na}}m_{\mathrm{M}}^3h_{\mathrm{M}}S_{\mathrm{M}}(E_{\mathrm{Na^+,M}}-V_{\mathrm{M}})$
$I_{\mathrm{NCX,M}}$	$\mathrm{Na^+}$-$\mathrm{Ca^{2+}}$ exchanger current	$g_{\mathrm{NCX}}Q_{\mathrm{NCX}}K_{\mathrm{a,NCX}}\frac{s_1-s_2}{s_3\left(1+k_{\mathrm{sat}}\exp\left(\frac{(\nu_{\mathrm{NCX}}-1)V_{\mathrm{M}}F}{RT}\right)\right)}$, where $s_1=\exp\left(\nu_{\mathrm{NCX}}V_M\frac{F}{RT}\right)[\mathrm{Na^+}]_{\mathrm{i}}^3[\mathrm{Ca^{2+}}]_{\mathrm{o}}$, $s_2=\exp\left((\nu_{\mathrm{NCX}}-1.0)\frac{V_{\mathrm{M}}F}{RT}\right)[\mathrm{Na^+}]_{\mathrm{o}}^3[\mathrm{Ca^{2+}}]_{\mathrm{i}}$, $s_3=K_{\mathrm{Ca,i}}[\mathrm{Na^+}]_{\mathrm{o}}{}^3\left(1+\left(\frac{[\mathrm{Na^+}]_{\mathrm{i}}}{K_{\mathrm{Na,i}}}\right)^3\right)+K_{\mathrm{Na,o}}^3[\mathrm{Ca^{2+}}]_{\mathrm{i}}\left(1+\frac{[\mathrm{Ca^{2+}}]_{\mathrm{i}}}{K_{\mathrm{Ca,i}}}\right)+K_{\mathrm{Ca,o}}[\mathrm{Na^+}]_{\mathrm{i}}^3+[\mathrm{Na^+}]_{\mathrm{i}}^3[\mathrm{Ca^{2+}}]_{\mathrm{o}}+[\mathrm{Na^+}]_{\mathrm{o}}^3[\mathrm{Ca^{2+}}]_{\mathrm{i}}$,

Continued on next page

bioRxiv preprint doi: https://doi.org/10.1101/2025.05.22.655415; this version posted July 24, 2026. The copyright holder for this preprint (which was not certified by peer review) is the author/funder, who has granted bioRxiv a license to display the preprint in perpetuity. It is made available under a CC-BY 4.0 International license.

Table A5: (continued) Reactions and fluxes

Name	Description	Equation
		and $K_{\text{a,NCX}} = \dfrac{1.0}{1.0 + \left(\dfrac{K_{\text{dAct}}}{[\text{Ca}^{2+}]_{\text{i}}}\right)^2}$
$I_{\text{Cl}^-,\text{M}}$	Cl^- channel current	$g_{\text{Cl}} Q_{\text{Cl}} A^4 (E_{\text{Cl}^-,\text{M}} - V_{\text{M}})$, where $A = \dfrac{1}{1 + \exp\left(\dfrac{V_{\text{M}} - V_{\text{a}}}{A_{\text{a}}}\right)}$
**Module 2**		
J_{RyR}	Ca^{2+} release through RyR	$\nu_{\text{max,RyR}} P_{\text{O,MWC}} w_{\text{RyR}} ([\text{Ca}^{2+}]_{\text{SR,junc}} - [\text{Ca}^{2+}]_{\text{myo,junc}})$ where $P_{\text{O,MWC}} = \dfrac{\left(1 + \epsilon_V f_{\text{RyR}}{}^{-2}\right)^4}{\left(1 + \epsilon_V f_{\text{RyR}}^{-2}\right)^4 + L_{\text{RyR}} (1 + \epsilon_V)^4}$ and $\epsilon_V = \exp\left(\dfrac{V_{\text{M}} - V_{\text{RyR}}}{4K_{\text{RyR}}}\right)$
**Module 3**		
$J_{\text{PMCA,M}}$	Ca^{2+} efflux through PMCA	$J_{\text{PMCA,0}} Q_{\text{PMCA}} \left(\dfrac{[\text{Ca}^{2+}]_{\text{i}}}{K_{\text{PMCA}} + [\text{Ca}^{2+}]_{\text{i}}}\right) \left(\dfrac{[\text{ATP}]_{\text{i}}}{k_{\text{ATP}} + [\text{ATP}]_{\text{i}}}\right)$
$I_{\text{Ca}^{2+}\text{leak,M}}$	Ca^{2+} leak through SRM	$g_{\text{leak,M}} ([\text{Ca}^{2+}]_{\text{o}} - [\text{Ca}^{2+}]_{\text{i}})$
J_{SERCA}	SERCA Ca^{2+} flux into SR	$J_{\text{SERCA,0}} Q_{\text{SERCA}} \left(\dfrac{[\text{Ca}^{2+}]_{\text{i}}}{K_{\text{SERCA}} + [\text{Ca}^{2+}]_{\text{i}}}\right) \left(\dfrac{[\text{ATP}]_{\text{i}}}{k_{\text{ATP}} + [\text{ATP}]_{\text{i}}}\right)$
$J_{\text{Ca}^{2+}\text{leak, SR}}$	Ca^{2+} leak through SRM	$\nu_{\text{leak,SR}} ([\text{Ca}^{2+}]_{\text{SR}} - [\text{Ca}^{2+}]_{\text{i}})$
J_{SOCE}	Ca^{2+} influx due to SOCE	$P_{\text{O,Orai1}} g_{\text{Orai1}} \dfrac{2F}{N_A} (E_{\text{Ca}^{2+},\text{junc}} - V_{\text{SL}})$
**Module 4**		

Continued on next page

bioRxiv preprint doi: https://doi.org/10.1101/2025.05.22.655415; this version posted July 24, 2026. The copyright holder for this preprint (which was not certified by peer review) is the author/funder, who has granted bioRxiv a license to display the preprint in perpetuity. It is made available under a CC-BY 4.0 International license.

Table A5: (continued) Reactions and fluxes

Name	Description	Equation
$J_{\mathrm{Ca^{2+}\text{-}Parv,i}}$	Ca^{2+} binding to parvalbumin	$k_{\mathrm{on,CP}}[\mathrm{Parv}]_{\mathrm{i}}[\mathrm{Ca^{2+}}]_{\mathrm{i}} - k_{\mathrm{on,CP}}[\mathrm{Ca^{2+}\text{-}Parv}]_{\mathrm{i}}$
$J_{\mathrm{Mg^{2+}\text{-}Parv,i}}$	Mg^{2+} binding to parvalbumin	$k_{\mathrm{on,MP}}[\mathrm{Parv}]_{\mathrm{i}}[\mathrm{Mg^{2+}}] - k_{\mathrm{off,MP}}[\mathrm{Mg^{2+}\text{-}Parv}]_{\mathrm{i}}$
$J_{\mathrm{Ca^{2+}\text{-}ATP,i}}$	Ca^{2+} binding to ATP	$k_{\mathrm{on,CA}}[\mathrm{ATP}]_{\mathrm{i}}[\mathrm{Ca^{2+}}]_{\mathrm{i}} - k_{\mathrm{off,CA}}[\mathrm{Ca^{2+}\text{-}ATP}]_{\mathrm{i}}$
$J_{\mathrm{Mg^{2+}\text{-}ATP,i}}$	Mg^{2+} binding to ATP	$k_{\mathrm{on,MA}}[\mathrm{ATP}]_{\mathrm{i}}[\mathrm{Mg^{2+}}] - k_{\mathrm{off,MA}}[\mathrm{ATP\text{-}Mg^{2+}}]_{\mathrm{i}}$
$J_{\mathrm{Trop\text{-}Ca^{2+},i}}$	Ca^{2+} binding to troponin	$k_{\mathrm{on,T1}}[\mathrm{Ca^{2+}}]_{\mathrm{i}}[\mathrm{Trop}]_{\mathrm{i}} - k_{\mathrm{off,T1}}[\mathrm{Trop\text{-}Ca^{2+}}]_{\mathrm{i}}$
$J_{\mathrm{Trop\text{-}2Ca^{2+},i}}$	Ca^{2+} binding to Ca^{2+}-troponin	$k_{\mathrm{on,T2}}[\mathrm{Ca^{2+}}]_{\mathrm{i}}[\mathrm{Trop\text{-}Ca^{2+}}]_{\mathrm{i}} + k_{\mathrm{off,T2}}[\mathrm{Trop\text{-}2Ca^{2+}}]_{\mathrm{i}}$
$J_{\mathrm{Ca^{2+}\text{-}CSQN}}$	Ca^{2+} binding to calsequestrin in SR	$\frac{k_{\mathrm{off,CSQN}}}{K_{\mathrm{CSQN}}}[\mathrm{Ca^{2+}}]_{\mathrm{SR}}[\mathrm{CSQN}]_{\mathrm{SR}} - k_{\mathrm{off,CSQN}}([\mathrm{CSQN}]_{\mathrm{tot}} - [\mathrm{CSQN}]_{\mathrm{SR}})$
$J_{\mathrm{Ca^{2+}\text{-}phos}}$	Ca^{2+} precipitation with phosphate in SR	$\mathcal{H}([\mathrm{Pi}]_{\mathrm{SR}}[\mathrm{Ca^{2+}}]_{\mathrm{SR}} - \mathrm{PP})([\mathrm{Pi}]_{\mathrm{SR}}[\mathrm{Ca^{2+}}]_{\mathrm{SR}} - \mathrm{PP})A_{\mathrm{P}}[\mathrm{Pi}]_{\mathrm{SR}}[\mathrm{Ca^{2+}}]_{\mathrm{SR}} - \mathcal{H}(\mathrm{PP} - [\mathrm{Pi}]_{\mathrm{SR}}[\mathrm{Ca^{2+}}]_{\mathrm{SR}})B_{\mathrm{P}}(\mathrm{PP} - [\mathrm{Pi}]_{\mathrm{SR}}[\mathrm{Ca^{2+}}]_{\mathrm{SR}})[\mathrm{Ca^{2+}\text{-}Pi}]_{\mathrm{SR}}$ where $\mathcal{H}$ is the Heaviside function
$J_{\mathrm{hyd,i}}$	total ATP hydrolysis rate	$\frac{N_{\mathrm{A}} I_{\mathrm{NaK,M}}}{F} + J_{\mathrm{PMCA,M}} + \frac{J_{\mathrm{SERCA,SR}} SA_{\mathrm{SR}}}{2 vol_{\mathrm{i}}} + k_{\mathrm{hyd}} \frac{[\mathrm{ATP}]_{\mathrm{i}}}{[\mathrm{ATP}]_{\mathrm{i}} + K_{\mathrm{ATP}}}$
$J_{\mathrm{phos,SR}}$	transport of phosphate into SR	$k_{\mathrm{P}}([\mathrm{Pi}]_{\mathrm{i}} - [\mathrm{Pi}]_{\mathrm{SR}})$
J_D	rate of tropomyosin unblocking	$k_{\mathrm{unblock}}[\mathrm{Trop\text{-}2Ca^{2+}}][\mathrm{ATP}]_{\mathrm{i}} - k_{\mathrm{block}}[D]$
J_{pre}	rate of myosin head binding	$f_0[D] - f_{\mathrm{p}}[A_{\mathrm{Pre}}]$
J_{post}	rate of power stroke	$h_0[A_{\mathrm{Pre}}] - h_{\mathrm{p}}[A_{\mathrm{Post}}][\mathrm{Pi}]_{\mathrm{i}}$
J_{detach}	rate of myosin head detaching post-power stroke	$g_0[A_{\mathrm{Post}}][\mathrm{ATP}]_{\mathrm{i}}$
**Module 5**		

Continued on next page

bioRxiv preprint doi: https://doi.org/10.1101/2025.05.22.655415; this version posted July 24, 2026. The copyright holder for this preprint (which was not certified by peer review) is the author/funder, who has granted bioRxiv a license to display the preprint in perpetuity. It is made available under a CC-BY 4.0 International license.

Table A5: (continued) Reactions and fluxes

Name	Description	Equation
J_{MCU}	rate of mitochondrial Ca^{2+} import through MCU	$\nu_{\mathrm{MCU}} \frac{\frac{[\mathrm{Ca}^{2+}]_{\mathrm{i}}}{K_{\mathrm{trans}}}\left(1+\frac{[\mathrm{Ca}^{2+}]_{\mathrm{i}}}{K_{\mathrm{trans}}}\right)^3}{\left(1+\frac{[\mathrm{Ca}^{2+}]_{\mathrm{i}}}{0K_{\mathrm{trans}}}\right)^4 + L_{\mathrm{MCU}}\left(1+\frac{[\mathrm{Ca}^{2+}]_{\mathrm{i}}}{K_{\mathrm{act}}}\right)^{-2.3}} \exp\left(p_1 V_{\mathrm{Mito}}\right)$
$J_{\mathrm{NCX,M}}$	rate of mitochondrial Ca^{2+} export through NCX	$\nu_{\mathrm{NCX,Mito}}\left(\frac{[\mathrm{Ca}^{2+}]_{\mathrm{Mito}}}{[\mathrm{Ca}^{2+}]_{\mathrm{i}}}\right)\exp\left(p_2 V_{\mathrm{Mito}}\right)$
J_{mPTP}	Ca^{2+} flux through mitochondrial Permeability Transition Pore	$k_{\mathrm{mPTP}}([\mathrm{Ca}^{2+}]_{\mathrm{i}} - [\mathrm{Ca}^{2+}]_{\mathrm{Mito}})\exp\left(p_3 V_{\mathrm{Mito}}\right)$
J_{AGC}	rate of NADH shuttling due to the aspartate-glutamate carrier	$\nu_{\mathrm{AGC}}\left(\frac{[\mathrm{Ca}^{2+}]_{\mathrm{i}}}{K_{\mathrm{AGC}}+[\mathrm{Ca}^{2+}]_{\mathrm{i}}}\right)\left(\frac{K_{\mathrm{iCa}}}{K_{\mathrm{iCa}}+[\mathrm{Ca}^{2+}]_{\mathrm{Mito}}}\right)$
J_{Gly}	NADH production rate due to glycolysis in the cytosol	$k_{\mathrm{Gly}}\frac{vol_{\mathrm{Mito}}}{vol_{\mathrm{myo}}}\frac{[\mathrm{ATP}]_{\mathrm{set}}-[\mathrm{ATP}]_{\mathrm{i}}}{10\ \mu\mathrm{M}}\frac{[\mathrm{Pi}]_{\mathrm{i}}}{1000\ \mu\mathrm{M}}$
J_{PDH}	NADH production rate due to glycolysis, pyruvate dehydrogenase, and Krebs cycle	$J_{\mathrm{Gly}}\left(\frac{vol_{\mathrm{myo}}}{vol_{\mathrm{Mito}}}\right)\left(\frac{[\mathrm{Ca}^{2+}]_{\mathrm{Mito}}}{K_{\mathrm{mCa}}+[\mathrm{Ca}^{2+}]_{\mathrm{Mito}}}\right)\left(\frac{1}{K_{\mathrm{mNAD}}+\frac{[\mathrm{NADH}]_{\mathrm{Mito}}}{[\mathrm{NAD}]_{\mathrm{tot}}-[\mathrm{NADH}]_{\mathrm{Mito}}}}\right)$
J_{Ox}	NADH oxidation rate coupled to proton extrusion from mitochondria	$\nu_{\mathrm{Ox}}\left(\frac{[\mathrm{NADH}]_{\mathrm{Mito}}}{K_{\mathrm{Ox}}+[\mathrm{NADH}]_{\mathrm{Mito}}}\right)\frac{1}{1+\exp\left(\frac{V_{\mathrm{Mito}}-p_4}{p_5}\right)}$
J_{F1FO}	ADP phosphorylation rate by the F1FO ATPases	$\nu_{\mathrm{F1FO}}\frac{1}{1+\exp\left(\frac{p_6-V_{\mathrm{Mito}}}{p_7}\right)}\left(\frac{K_{\mathrm{iATP}}}{K_{\mathrm{iATP}}+[\mathrm{ATP}]_{\mathrm{Mito}}}\right)\left(\frac{[\mathrm{Pi}]_{\mathrm{Mito}}}{1000\ \mu\mathrm{M}}\right)$
J_{ANT}	rate of mitochondrial ADP import / ATP export	$\nu_{\mathrm{ANT}}\frac{1-\left(\frac{\alpha_c[\mathrm{ATP}]_{\mathrm{i,tot}}}{[\mathrm{ADP}]_{\mathrm{i}}}\right)\left(\frac{[\mathrm{ANP}]_{\mathrm{tot}}-[\mathrm{ATP}]_{\mathrm{Mito}}}{\alpha_{\mathrm{Mito}}[\mathrm{ATP}]_{\mathrm{Mito}}}\right)\exp\left(-\frac{FV_{\mathrm{Mito}}}{RT}\right)}{\left(1+\frac{\alpha_c[\mathrm{ATP}]_{\mathrm{i,tot}}}{[\mathrm{ADP}]_{\mathrm{i}}}\exp\left(-f_{\mathrm{ANT}}\frac{FV_{\mathrm{Mito}}}{RT}\right)\right)\left(1+\frac{[\mathrm{ANP}]_{\mathrm{tot}}-[\mathrm{ATP}]_{\mathrm{Mito}}}{\alpha_{\mathrm{Mito}}[\mathrm{ATP}]_{\mathrm{Mito}}}\right)}$ where $[\mathrm{ATP}]_{\mathrm{i,tot}} = [\mathrm{ATP}]_{\mathrm{i}} + [\mathrm{Ca}^{2+}\text{-ATP}]_{\mathrm{i,tot}} + [\mathrm{Mg}^{2+}\text{-ATP}]_{\mathrm{i}}$

Continued on next page

bioRxiv preprint doi: https://doi.org/10.1101/2025.05.22.655415; this version posted July 24, 2026. The copyright holder for this preprint (which was not certified by peer review) is the author/funder, who has granted bioRxiv a license to display the preprint in perpetuity. It is made available under a CC-BY 4.0 International license.

Table A5: (continued) Reactions and fluxes

Name	Description	Equation
$J_{\text{Hleak,M}}$	rate of mitochondrial proton leak	$p_8 V_{\text{Mito}} + p_9$
J_{PiC}	rate of phosphate transport into mitochondria	$\nu_{\text{PiC}} \dfrac{\frac{[\text{Pi}]_{\text{myo}}}{K_{\text{Pi}}} - \frac{[\text{Pi}]_{\text{Mito}}}{K_{\text{Pi,M}}}}{1 + \frac{[\text{Pi}]_{\text{myo}}}{K_{\text{Pi}}} + \frac{[\text{Pi}]_{\text{Mito}}}{K_{\text{Pi,M}}}}$
$J_{\text{Ca}^{2+}\text{-phos,M}}$	rate of Ca^{2+}-phosphate binding in the mitochondria	$\dfrac{k_{\text{off,B}}}{K_{\text{dB}}}[\text{Pi}]_{\text{Mito}}[\text{Ca}^{2+}]_{\text{Mito}} - k_{\text{off,B}}[\text{Ca}^{2+}\text{-Pi}]_M$

Table A6: System of ODEs.

Variable	Equation
**Junctional variables**	
$\dfrac{d[\text{Na}^+]_{\text{TT}}}{dt}$	$\dfrac{SA_{\text{TT}}\eta_{\text{conv,1}}}{vol_{\text{TT}}F}(-0.1I_{\text{Na}^+,\text{TT}} + 3\times 0.1I_{\text{NaK,TT}} - I_{\text{NCX,TT}}) + \dfrac{D_{\text{ion,EC}}SA_{\text{EC,bulk-junc}}}{vol_{\text{TT}}\Delta d_{\text{diff}}}([\text{Na}^+]_{\text{EC}} - [\text{Na}^+]_{\text{TT}})$
$\dfrac{d[\text{Na}^+]_{\text{myo,junc}}}{dt}$	$\dfrac{SA_{\text{TT}}\eta_{\text{conv,1}}}{vol_{\text{myo,junc}}F}(0.1I_{\text{Na}^+,\text{TT}} - 3\times 0.1I_{\text{NaK,TT}} + I_{\text{NCX,TT}}) + \dfrac{D_{\text{ion}}SA_{\text{myo,bulk-junc}}}{vol_{\text{myo,junc}}\Delta d_{\text{diff}}}([\text{Na}^+]_{\text{myo,bulk}} - [\text{Na}^+]_{\text{myo,junc}})$
$\dfrac{dm_{\text{TT}}}{dt}$	$\alpha_m(1 - m_{\text{TT}}) - \beta_m m_{\text{TT}}$, where $\beta_m = \beta_{m0}Q_{\beta_m}\exp\left(-\dfrac{V_{\text{TT}} - V_m}{K_{\beta m}}\right)$ and $\alpha_m = \alpha_{m0}Q_{\alpha_m}\dfrac{V_{\text{TT}} - V_m}{1 - \exp\left(\dfrac{V_m - V_{\text{TT}}}{K_{\alpha_m}}\right)}$

Continued on next page

bioRxiv preprint doi: https://doi.org/10.1101/2025.05.22.655415; this version posted July 24, 2026. The copyright holder for this preprint (which was not certified by peer review) is the author/funder, who has granted bioRxiv a license to display the preprint in perpetuity. It is made available under a CC-BY 4.0 International license.

Table A6: (continued) System of ODEs.

Variable	Equation
$\frac{dh_{\text{TT}}}{dt}$	$\alpha_h(1-h_{\text{TT}})-(\beta_h h_{\text{TT}})$, where $\beta_h = Q_{\beta_h}\frac{\beta_{h0}}{1+\exp\left(-\frac{V_{\text{TT}}-V_h}{K_{\beta_h}}\right)}$ and $\alpha_h=\alpha_{h0}Q_{\alpha_h}\exp\left(-\frac{V_{\text{TT}}-V_h}{K_{\alpha_h}}\right)$
$\frac{dS_{\text{TT}}}{dt}$	$\frac{S_\infty - S_{\text{TT}}}{\tau_S}$, where $S_\infty = \frac{1}{1+\exp\left(\frac{V_{\text{TT}}-V_{S\infty}}{A_{S\infty}}\right)}$ and $\tau_S = \frac{60}{0.2+5.65\left(\frac{V_{\text{TT}}+V_\tau}{100}\right)^2}$
$\frac{d[\text{K}^+]_{\text{TT}}}{dt}$	$\frac{SA_{\text{TT}}\eta_{\text{conv},1}}{vol_{\text{TT}}F}(-I_{\text{KIR,TT}}+0.45I_{\text{KDR,TT}}-2\times 0.1I_{\text{NaK,TT}})+\frac{D_{\text{ion,EC}}SA_{\text{EC,bulk-junc}}}{vol_{\text{TT}}\Delta d_{\text{diff}}}([\text{K}^+]_{\text{EC}}-[\text{K}^+]_{\text{TT}})$
$\frac{d[\text{K}^+]_{\text{myo,junc}}}{dt}$	$\frac{SA_{\text{TT}}\eta_{\text{conv},1}}{vol_{\text{myo,junc}}F}(I_{\text{KIR,TT}}-0.45I_{\text{KDR,TT}}+2\times 0.1I_{\text{NaK,TT}})+\frac{D_{\text{ion}}SA_{\text{myo,bulk-junc}}}{vol_{\text{myo,junc}}\Delta d_{\text{diff}}}([\text{K}^+]_{\text{myo,bulk}}-[\text{K}^+]_{\text{myo,junc}})$
$\frac{dn_{\text{TT}}}{dt}$	$\alpha_n(1-n_{\text{TT}})-\beta_n n_{\text{TT}}$, where $\beta_n=\beta_{n0}Q_{\beta_n}\exp\left(-\frac{V_{\text{TT}}-V_n}{K_{\beta_n}}\right)$ and $\alpha_n=\alpha_{n0}Q_{\alpha_n}\frac{V_{\text{TT}}-V_n}{1-\exp\left(\frac{V_n-V_{\text{TT}}}{K_{\alpha_n}}\right)}$
$\frac{dh_{\text{K,TT}}}{dt}$	$\frac{h_{\text{K}\infty}-h_{\text{K,TT}}}{\tau_{h_{\text{K}}}}$, where $\tau_{h_{\text{K}}}=\exp\left(-\frac{V_{\text{TT}}+40}{25.75}\right)$ and $h_{\text{K}\infty}=\left(1+\exp\left(\frac{V_{\text{TT}}-V_{h_{\text{K}\infty}}}{A_{h_{\text{K}\infty}}}\right)\right)^{-1}$
$\frac{d[\text{Cl}^-]_{\text{TT}}}{dt}$	$-\frac{SA_{\text{TT}}\eta_{\text{conv},1}}{vol_{\text{TT}}F}(-0.1I_{\text{Cl}^-,\text{TT}})+\frac{D_{\text{ion,EC}}SA_{\text{EC,bulk-junc}}}{vol_{\text{TT}}\Delta d_{\text{diff}}}([\text{Cl}^-]_{\text{EC}}-[\text{Cl}^-]_{\text{TT}})$

Continued on next page

bioRxiv preprint doi: https://doi.org/10.1101/2025.05.22.655415; this version posted July 24, 2026. The copyright holder for this preprint (which was not certified by peer review) is the author/funder, who has granted bioRxiv a license to display the preprint in perpetuity. It is made available under a CC-BY 4.0 International license.

Table A6: (continued) System of ODEs.

Variable	Equation
$\frac{d[\text{Cl}^-]_{\text{myo,junc}}}{dt}$	$-\frac{SA_{\text{TT}}\eta_{\text{conv},1}}{vol_{\text{myo,junc}}F}(0.1I_{\text{Cl}^-,\text{TT}}) + \frac{D_{\text{ion}}SA_{\text{myo,bulk-junc}}}{vol_{\text{myo,junc}}\Delta d_{\text{diff}}}([\text{Cl}^-]_{\text{myo,bulk}} - [\text{Cl}^-]_{\text{myo,junc}})$
$\frac{d[\text{Ca}^{2+}]_{\text{TT}}}{dt}$	$\frac{SA_{\text{TT}}}{vol_{\text{TT}}\eta_{\text{conv},2}}(-J_{\text{Ca}^{2+}\text{ leak,TT}} - J_{\text{SOCE,TT}} + \frac{N_{\text{A}}}{F}I_{\text{NCX,TT}}) + J_{\text{PMCA,TT}}\frac{vol_{\text{myo,junc}}}{vol_{\text{TT}}} + \frac{D_{\text{ion,EC}}SA_{\text{EC,bulk-junc}}}{vol_{\text{TT}}\Delta d_{\text{diff}}}([\text{Ca}^{2+}]_{\text{EC}} - [\text{Ca}^{2+}]_{\text{TT}})$
$\frac{d[\text{Ca}^{2+}]_{\text{myo,junc}}}{dt}$	$\frac{SA_{\text{TT}}}{vol_{\text{myo,junc}}\eta_{\text{conv},2}}(J_{\text{Ca}^{2+}\text{ leak,TT}} + J_{\text{SOCE,TT}} - \frac{N_{\text{A}}}{F}I_{\text{NCX,TT}}) - J_{\text{PMCA,TT}} + \frac{SA_{\text{SRM,junc}}}{vol_{\text{SR,junc}}}(J_{\text{RyR,junc}} + J_{\text{Ca}^{2+}\text{ leak SR,junc}} - 0.1J_{\text{SERCA,junc}}) - J_{\text{Ca}^{2+}\text{-Parv,junc}} - J_{\text{Ca}^{2+}\text{-ATP,junc}} - \frac{vol_{\text{Mito,junc}}}{vol_{\text{myo,junc}}}(J_{\text{MCU,junc}} - J_{\text{NCX,Mito,junc}} + J_{\text{mPTP,junc}}) + \frac{D_{\text{ion}}SA_{\text{myo,bulk-junc}}}{vol_{\text{myo,junc}}\Delta d_{\text{diff}}}([\text{Ca}^{2+}]_{\text{myo,bulk}} - [\text{Ca}^{2+}]_{\text{myo,junc}})$
$\frac{d[\text{Ca}^{2+}]_{\text{SR,junc}}}{dt}$	$\frac{SA_{\text{SRM,junc}}}{vol_{\text{SR,junc}}}(-J_{\text{Ca}^{2+}\text{ leak SR,junc}} - J_{\text{RyR,junc}} + 0.1J_{\text{SERCA,junc}}) - J_{\text{Ca}^{2+}\text{-CSQN,junc}} + \frac{D_{\text{ion}}SA_{\text{SR,bulk-junc}}}{vol_{\text{SR,junc}}\Delta d_{\text{diff}}}([\text{Ca}^{2+}]_{\text{SR,bulk}} - [\text{Ca}^{2+}]_{\text{SR,junc}})$
$\frac{dw_{\text{RyR}}}{dt}$	$\alpha_w\left(1 - w_{\text{RyR}}\left(1 + \frac{[\text{Ca}^{2+}]_{\text{myo,junc}}}{K_w}\right)\right)$
$\frac{d[\text{Ca}^{2+}\text{-Parv}]_{\text{myo,junc}}}{dt}$	$J_{\text{Ca}^{2+}\text{-Parv,junc}} + \frac{D_{\text{Parv}}SA_{\text{myo,bulk-junc}}}{vol_{\text{myo,junc}}\Delta d_{\text{diff}}}([\text{Ca}^{2+}\text{-Parv}]_{\text{myo,bulk}} - [\text{Ca}^{2+}\text{-Parv}]_{\text{myo,junc}})$
$\frac{d[\text{Mg}^{2+}\text{-Parv}]_{\text{myo,junc}}}{dt}$	$J_{\text{Mg}^{2+}\text{-Parv,junc}} + \frac{D_{\text{Parv}}SA_{\text{myo,bulk-junc}}}{vol_{\text{myo,junc}}\Delta d_{\text{diff}}}([\text{Mg}^{2+}\text{-Parv}]_{\text{myo,bulk}} - [\text{Mg}^{2+}\text{-Parv}]_{\text{myo,junc}})$

Continued on next page

bioRxiv preprint doi: https://doi.org/10.1101/2025.05.22.655415; this version posted July 24, 2026. The copyright holder for this preprint (which was not certified by peer review) is the author/funder, who has granted bioRxiv a license to display the preprint in perpetuity. It is made available under a CC-BY 4.0 International license.

Table A6: (continued) System of ODEs.

Variable	Equation
$\frac{d[\text{ATP}]_{\text{myo,junc}}}{dt}$	$-J_{\text{Ca}^{2+}\text{-ATP,junc}} - J_{\text{Mg}^{2+}\text{-ATP,junc}} - J_{\text{hydro,junc}} + J_{\text{Gly,junc}} N_{\text{ATP-per-NADH}} + J_{\text{ANT,junc}} \frac{vol_{\text{Mito,junc}}}{vol_{\text{myo,junc}}} + \frac{D_{\text{ATP}} SA_{\text{myo,bulk-junc}}}{vol_{\text{myo,junc}} \Delta d_{\text{diff}}} ([\text{ATP}]_{\text{myo,bulk}} - [\text{ATP}]_{\text{myo,junc}})$
$\frac{d[\text{Ca}^{2+}\text{-ATP}]_{\text{myo,junc}}}{dt}$	$J_{\text{Ca}^{2+}\text{-ATP,junc}} + \frac{D_{\text{ATP}} SA_{\text{myo,bulk-junc}}}{vol_{\text{myo,junc}} \Delta d_{\text{diff}}} ([\text{Ca}^{2+}\text{-ATP}]_{\text{myo,bulk}} - [\text{Ca}^{2+}\text{-ATP}]_{\text{myo,junc}})$
$\frac{d[\text{Mg}^{2+}\text{-ATP}]_{\text{myo,junc}}}{dt}$	$J_{\text{Mg}^{2+}\text{-ATP,junc}} + \frac{D_{\text{ATP}} SA_{\text{myo,bulk-junc}}}{vol_{\text{myo,junc}} \Delta d_{\text{diff}}} ([\text{Mg}^{2+}\text{-ATP}]_{\text{myo,bulk}} - [\text{Mg}^{2+}\text{-ATP}]_{\text{myo,junc}})$
$\frac{d[\text{Pi}]_{\text{myo,junc}}}{dt}$	$J_{\text{hydro,junc}} - J_{\text{Gly,junc}} N_{\text{ATP-per-NADH}} - J_{\text{phos,SR,junc}} - J_{\text{PiC,junc}} \frac{vol_{\text{Mito,junc}}}{vol_{\text{myo,junc}}} + \frac{D_{\text{Pi}} SA_{\text{myo,bulk-junc}}}{vol_{\text{myo,junc}} \Delta d_{\text{diff}}} ([\text{Pi}]_{\text{myo,bulk}} - [\text{Pi}]_{\text{myo,junc}})$
$\frac{d[\text{Pi}]_{\text{SR,junc}}}{dt}$	$J_{\text{phos,SR,junc}} - J_{\text{Ca}^{2+}\text{-phos,junc}} + \frac{D_{\text{Pi}} SA_{\text{SR,bulk-junc}}}{vol_{\text{SR,junc}} \Delta d_{\text{diff}}} ([\text{Pi}]_{\text{SR,bulk}} - [\text{Pi}]_{\text{SR,junc}})$
$\frac{d[\text{Ca}^{2+}\text{-Pi}]_{\text{SR,junc}}}{dt}$	$J_{\text{Ca}^{2+}\text{-phos,junc}} + \frac{D_{\text{Pi}} SA_{\text{SR,bulk-junc}}}{vol_{\text{SR,junc}} \Delta d_{\text{diff}}} ([\text{Ca}^{2+}\text{-Pi}]_{\text{SR,bulk}} - [\text{Ca}^{2+}\text{-Pi}]_{\text{SR,junc}})$
$\frac{d[\text{CSQN}]_{\text{SR,junc}}}{dt}$	$-J_{\text{Ca}^{2+}\text{-CSQN,junc}} + \frac{D_{\text{CSQN}} SA_{\text{SR,bulk-junc}}}{vol_{\text{SR,junc}} \Delta d_{\text{diff}}} ([\text{CSQN}]_{\text{SR,bulk}} - [\text{CSQN}]_{\text{SR,junc}})$
$\frac{d[\text{ADP}]_{\text{myo,junc}}}{dt}$	$J_{\text{hydro,junc}} - J_{\text{Gly,junc}} N_{\text{ATP-per-NADH}} - J_{\text{ANT,junc}} \frac{vol_{\text{Mito,junc}}}{vol_{\text{myo,junc}}} + \frac{D_{\text{ATP}} SA_{\text{myo,bulk-junc}}}{vol_{\text{myo,junc}} \Delta d_{\text{diff}}} ([\text{ADP}]_{\text{myo,bulk}} - [\text{ADP}]_{\text{myo,junc}})$

Continued on next page

bioRxiv preprint doi: https://doi.org/10.1101/2025.05.22.655415; this version posted July 24, 2026. The copyright holder for this preprint (which was not certified by peer review) is the author/funder, who has granted bioRxiv a license to display the preprint in perpetuity. It is made available under a CC-BY 4.0 International license.

Table A6: (continued) System of ODEs.

Variable	Equation
$\frac{d[\text{Ca}^{2+}]_{\text{Mito,junc}}}{dt}$	$b_{\text{M}}\left(J_{\text{MCU,junc}} - J_{\text{NCX,Mito,junc}} + J_{\text{mPTP,junc}} - J_{\text{Ca}^{2+}\text{-phos,Mito,junc}}\right)$
$\frac{d[\text{Pi}]_{\text{Mito,junc}}}{dt}$	$J_{\text{PiC,junc}} - J_{\text{Ca}^{2+}\text{-phos,Mito,junc}}$
$\frac{d[\text{Ca}^{2+}\text{-Pi}]_{\text{Mito,junc}}}{dt}$	$J_{\text{Ca}^{2+}\text{-phos,Mito,junc}}$
$\frac{d[\text{ATP}]_{\text{Mito,junc}}}{dt}$	$J_{\text{F1FO,junc}} - J_{\text{ANT,junc}}$
$\frac{d[\text{NADH}]_{\text{Mito,junc}}}{dt}$	$J_{\text{PDH,junc}} + J_{\text{AGC,junc}} - J_{\text{Ox,junc}}$
$\frac{dV_{\text{Mito,junc}}}{dt}$	$(a_1 J_{\text{Ox,junc}} - a_2 J_{\text{F1FO,junc}} - J_{\text{Hleak,Mito,junc}} - J_{\text{NCX,Mito,junc}} - 2J_{\text{MCU,junc}} - 2J_{\text{mPTP,junc}} - J_{\text{ANT,junc}} - J_{\text{AGC,junc}})/C_{\text{Eff,Mito}}$
**Bulk variables**	
$\frac{d[\text{Na}^+]_{\text{myo,bulk}}}{dt}$	$\frac{SA_{\text{SL}}\eta_{\text{conv,1}}}{vol_{\text{myo,bulk}}F}(I_{\text{Na}^+\text{,SL}} - 3I_{\text{NaK,SL}} + I_{\text{NCX,SL}}) + \frac{D_{\text{ion}}SA_{\text{myo,bulk-junc}}}{vol_{\text{myo,bulk}}\Delta d_{\text{diff}}}([\text{Na}^+]_{\text{myo,junc}} - [\text{Na}^+]_{\text{myo,bulk}})$
$\frac{dm_{\text{SL}}}{dt}$	$\frac{SA_{\text{SL}}\eta_{\text{conv,1}}}{vol_{\text{myo,bulk}}F}(I_{\text{KIR,SL}} - I_{\text{KDR,SL}} + 2I_{\text{NaK,TT}}) + \frac{D_{\text{ion}}SA_{\text{myo,bulk-junc}}}{vol_{\text{myo,bulk}}\Delta d_{\text{diff}}}([\text{K}^+]_{\text{myo,junc}} - [\text{K}^+]_{\text{myo,bulk}})$

Continued on next page

bioRxiv preprint doi: https://doi.org/10.1101/2025.05.22.655415; this version posted July 24, 2026. The copyright holder for this preprint (which was not certified by peer review) is the author/funder, who has granted bioRxiv a license to display the preprint in perpetuity. It is made available under a CC-BY 4.0 International license.

Table A6: (continued) System of ODEs.

Variable	Equation
$\frac{dh_{\text{SL}}}{dt}$	$\alpha_h(1-h_{\text{SL}})-(\beta_h h_{\text{SL}})$, where $\beta_h = Q_{\beta_h}\frac{\beta_{h0}}{1+\exp\left(-\frac{V_{\text{SL}}-V_h}{K_{\beta_h}}\right)}$ and $\alpha_h = \alpha_{h0}Q_{\alpha_h}\exp\left(-\frac{V_{\text{SL}}-V_h}{K_{\alpha_h}}\right)$
$\frac{dS_{\text{SL}}}{dt}$	$\frac{S_\infty - S_{\text{SL}}}{\tau_S}$, where $S_\infty = \frac{1}{1+\exp\left(\frac{V_{\text{SL}}-V_{S\infty}}{A_{S\infty}}\right)}$ and $\tau_S = \frac{60}{0.2+5.65\left(\frac{V_{\text{SL}}+V_\tau}{100}\right)^2}$
$\frac{d[\text{K}^+]_{\text{myo,bulk}}}{dt}$	$\frac{SA_{\text{SL}}\eta_{\text{conv,1}}}{vol_{\text{myo,bulk}}F}(I_{\text{KIR,SL}} - I_{\text{KDR,SL}} + 2I_{\text{NaK,SL}}) + \frac{D_{\text{ion}}SA_{\text{myo,bulk-junc}}}{vol_{\text{myo,bulk}}\Delta d_{\text{diff}}}([\text{K}^+]_{\text{myo,junc}} - [\text{K}^+]_{\text{myo,bulk}})$
$\frac{dn_{\text{SL}}}{dt}$	$\alpha_n(1-n_{\text{SL}})-\beta_n n_{\text{SL}}$, where $\beta_n = \beta_{n0}Q_{\beta_n}\exp\left(-\frac{V_{\text{SL}}-V_n}{K_{\beta_n}}\right)$ and $\alpha_n = \alpha_{n0}Q_{\alpha_n}\frac{V_{\text{SL}}-V_n}{1-\exp\left(\frac{V_n-V_{\text{SL}}}{K_{\alpha_n}}\right)}$
$\frac{dh_{\text{K,SL}}}{dt}$	$\frac{h_{\text{K}\infty}-h_{\text{K,SL}}}{\tau_{h_{\text{K}}}}$, where $\tau_{h_{\text{K}}} = \exp\left(-\frac{V_{\text{SL}}+40}{25.75}\right)$ and $h_{\text{K}\infty} = \left(1+\exp\left(\frac{V_{\text{SL}}-V_{h_{\text{K}\infty}}}{A_{h_{\text{K}\infty}}}\right)\right)^{-1}$
$\frac{d[\text{Cl}^-]_{\text{myo,bulk}}}{dt}$	$-\frac{SA_{\text{SL}}\eta_{\text{conv,1}}}{vol_{\text{myo,bulk}}F}(I_{\text{Cl}^-\text{,SL}}) + \frac{D_{\text{ion}}SA_{\text{myo,bulk-junc}}}{vol_{\text{myo,bulk}}\Delta d_{\text{diff}}}([\text{Cl}^-]_{\text{myo,junc}} - [\text{Cl}^-]_{\text{myo,bulk}})$

Continued on next page

bioRxiv preprint doi: https://doi.org/10.1101/2025.05.22.655415; this version posted July 24, 2026. The copyright holder for this preprint (which was not certified by peer review) is the author/funder, who has granted bioRxiv a license to display the preprint in perpetuity. It is made available under a CC-BY 4.0 International license.

Table A6: (continued) System of ODEs.

Variable	Equation
$\frac{d[\mathrm{Ca}^{2+}]_{\mathrm{myo,bulk}}}{dt}$	$\frac{SA_{\mathrm{SL}}}{vol_{\mathrm{myo,bulk}}\eta_{\mathrm{conv,2}}}(J_{\mathrm{Ca^{2+}\ leak,SL}} - \frac{N_{\mathrm{A}}}{F}I_{\mathrm{NCX,SL}}) - J_{\mathrm{PMCA,SL}} + \frac{SA_{\mathrm{SRM,bulk}}}{vol_{\mathrm{myo,bulk}}}(J_{\mathrm{Ca^{2+}\ leak\ SR,bulk}} - J_{\mathrm{SERCA,bulk}}) - J_{\mathrm{Ca^{2+}\text{-}Parv,bulk}} - J_{\mathrm{Ca^{2+}\text{-}ATP,bulk}} - \frac{vol_{\mathrm{Mito,bulk}}}{vol_{\mathrm{myo,bulk}}}(J_{\mathrm{MCU,bulk}} - J_{\mathrm{NCX,bulk}} + J_{\mathrm{mPTP,bulk}}) + \frac{D_{\mathrm{ion}}SA_{\mathrm{myo,bulk\text{-}junc}}}{vol_{\mathrm{myo,bulk}}\Delta d_{\mathrm{diff}}}([\mathrm{Ca}^{2+}]_{\mathrm{myo,junc}} - [\mathrm{Ca}^{2+}]_{\mathrm{myo,bulk}})$
$\frac{d[\mathrm{Ca}^{2+}]_{\mathrm{SR,bulk}}}{dt}$	$\frac{SA_{\mathrm{SRM,bulk}}}{vol_{\mathrm{SR,bulk}}}(-J_{\mathrm{Ca^{2+}\ leak\ SR,bulk}} + J_{\mathrm{SERCA,bulk}}) - J_{\mathrm{Ca^{2+}\text{-}CSQN,bulk}} + \frac{D_{\mathrm{ion}}SA_{\mathrm{SR,bulk\text{-}junc}}}{vol_{\mathrm{SR,bulk}}\Delta d_{\mathrm{diff}}}([\mathrm{Ca}^{2+}]_{\mathrm{SR,junc}} - [\mathrm{Ca}^{2+}]_{\mathrm{SR,bulk}})$
$\frac{d[\mathrm{Ca}^{2+}\text{-Parv}]_{\mathrm{myo,bulk}}}{dt}$	$J_{\mathrm{Ca^{2+}\text{-}Parv,bulk}} + \frac{D_{\mathrm{Parv}}SA_{\mathrm{myo,bulk\text{-}junc}}}{vol_{\mathrm{myo,bulk}}\Delta d_{\mathrm{diff}}}([\mathrm{Ca}^{2+}\text{-Parv}]_{\mathrm{myo,junc}} - [\mathrm{Ca}^{2+}\text{-Parv}]_{\mathrm{myo,bulk}})$
$\frac{d[\mathrm{Mg}^{2+}\text{-Parv}]_{\mathrm{myo,bulk}}}{dt}$	$J_{\mathrm{Mg^{2+}\text{-}Parv,bulk}} + \frac{D_{\mathrm{Parv}}SA_{\mathrm{myo,bulk\text{-}junc}}}{vol_{\mathrm{myo,bulk}}\Delta d_{\mathrm{diff}}}([\mathrm{Mg}^{2+}\text{-Parv}]_{\mathrm{myo,junc}} - [\mathrm{Mg}^{2+}\text{-Parv}]_{\mathrm{myo,bulk}})$
$\frac{d[\mathrm{ATP}]_{\mathrm{myo,bulk}}}{dt}$	$-J_{\mathrm{Ca^{2+}\text{-}ATP,bulk}} - J_{\mathrm{Mg^{2+}\text{-}ATP,bulk}} - J_{\mathrm{hydro,bulk}} + J_{\mathrm{Gly,bulk}}N_{\mathrm{ATP\text{-}per\text{-}NADH}} + J_{\mathrm{ANT,bulk}}\frac{vol_{\mathrm{Mito,bulk}}}{vol_{\mathrm{myo,bulk}}} - J_D - J_{\mathrm{detach}} + \frac{D_{\mathrm{ATP}}SA_{\mathrm{myo,bulk\text{-}junc}}}{vol_{\mathrm{myo,bulk}}\Delta d_{\mathrm{diff}}}([\mathrm{ATP}]_{\mathrm{myo,junc}} - [\mathrm{ATP}]_{\mathrm{myo,bulk}})$
$\frac{d[\mathrm{Ca}^{2+}\text{-ATP}]_{\mathrm{myo,bulk}}}{dt}$	$J_{\mathrm{Ca^{2+}\text{-}ATP,bulk}} + \frac{D_{\mathrm{ATP}}SA_{\mathrm{myo,bulk\text{-}junc}}}{vol_{\mathrm{myo,bulk}}\Delta d_{\mathrm{diff}}}([\mathrm{Ca}^{2+}\text{-ATP}]_{\mathrm{myo,junc}} - [\mathrm{Ca}^{2+}\text{-ATP}]_{\mathrm{myo,bulk}})$
$\frac{d[\mathrm{Mg}^{2+}\text{-ATP}]_{\mathrm{myo,bulk}}}{dt}$	$J_{\mathrm{Mg^{2+}\text{-}ATP,bulk}} + \frac{D_{\mathrm{ATP}}SA_{\mathrm{myo,bulk\text{-}junc}}}{vol_{\mathrm{myo,bulk}}\Delta d_{\mathrm{diff}}}([\mathrm{Mg}^{2+}\text{-ATP}]_{\mathrm{myo,junc}} - [\mathrm{Mg}^{2+}\text{-ATP}]_{\mathrm{myo,bulk}})$

Continued on next page

bioRxiv preprint doi: https://doi.org/10.1101/2025.05.22.655415; this version posted July 24, 2026. The copyright holder for this preprint (which was not certified by peer review) is the author/funder, who has granted bioRxiv a license to display the preprint in perpetuity. It is made available under a CC-BY 4.0 International license.

Table A6: (continued) System of ODEs.

Variable	Equation
$\frac{d[\mathrm{Pi}]_{\mathrm{myo,bulk}}}{dt}$	$J_{\mathrm{hydro,bulk}} - J_{\mathrm{phos,SR,bulk}} + J_{\mathrm{post}} - J_{\mathrm{PiC,bulk}} \frac{vol_{\mathrm{Mito,bulk}}}{vol_{\mathrm{myo,bulk}}} + \frac{D_{\mathrm{Pi}} SA_{\mathrm{myo,bulk\text{-}junc}}}{vol_{\mathrm{myo,bulk}} \Delta d_{\mathrm{diff}}} ([\mathrm{Pi}]_{\mathrm{myo,junc}} - [\mathrm{Pi}]_{\mathrm{myo,bulk}})$
$\frac{d[\mathrm{Pi}]_{\mathrm{SR,bulk}}}{dt}$	$J_{\mathrm{phos,SR,bulk}} - J_{\mathrm{Ca^{2+}\text{-}phos,bulk}} + \frac{D_{\mathrm{Pi}} SA_{\mathrm{SR,bulk\text{-}junc}}}{vol_{\mathrm{SR,bulk}} \Delta d_{\mathrm{diff}}} ([\mathrm{Pi}]_{\mathrm{SR,junc}} - [\mathrm{Pi}]_{\mathrm{SR,bulk}})$
$\frac{d[\mathrm{Ca^{2+}\text{-}Pi}]_{\mathrm{SR,bulk}}}{dt}$	$J_{\mathrm{Ca^{2+}\text{-}phos,bulk}} + \frac{D_{\mathrm{Pi}} SA_{\mathrm{SR,bulk\text{-}junc}}}{vol_{\mathrm{SR,bulk}} \Delta d_{\mathrm{diff}}} ([\mathrm{Ca^{2+}\text{-}Pi}]_{\mathrm{SR,junc}} - [\mathrm{Ca^{2+}\text{-}Pi}]_{\mathrm{SR,bulk}})$
$\frac{d[\mathrm{CSQN}]_{\mathrm{SR,bulk}}}{dt}$	$-J_{\mathrm{Ca^{2+}\text{-}CSQN,bulk}} + \frac{D_{\mathrm{CSQN}} SA_{\mathrm{SR,bulk\text{-}junc}}}{vol_{\mathrm{SR,bulk}} \Delta d_{\mathrm{diff}}} ([\mathrm{CSQN}]_{\mathrm{SR,junc}} - [\mathrm{CSQN}]_{\mathrm{SR,bulk}})$
$\frac{d[\mathrm{Ca^{2+}Trop}]}{dt}$	$J_{\mathrm{Trop\text{-}Ca^{2+}}} - J_{\mathrm{Trop\text{-}2Ca^{2+}}}$
$\frac{d[\mathrm{Trop\text{-}2Ca^{2+}}]}{dt}$	$J_{\mathrm{Trop\text{-}2Ca^{2+}}} - J_D$
$\frac{d[D]}{dt}$	$J_D - J_{\mathrm{pre}} + J_{\mathrm{detach}}$
$\frac{d[A_{\mathrm{pre}}]}{dt}$	$J_{\mathrm{pre}} - J_{\mathrm{post}}$
$\frac{d[A_{\mathrm{post}}]}{dt}$	$J_{\mathrm{post}} - J_{\mathrm{detach}}$

Continued on next page

bioRxiv preprint doi: https://doi.org/10.1101/2025.05.22.655415; this version posted July 24, 2026. The copyright holder for this preprint (which was not certified by peer review) is the author/funder, who has granted bioRxiv a license to display the preprint in perpetuity. It is made available under a CC-BY 4.0 International license.

Table A6: (continued) System of ODEs.

Variable	Equation
$\frac{d[\mathrm{ADP}]_{\mathrm{myo,bulk}}}{dt}$	$J_{\mathrm{hydro,bulk}} - J_{\mathrm{Gly,bulk}} N_{\mathrm{ATP\text{-}per\text{-}NADH}} - J_{\mathrm{ANT,bulk}} \frac{vol_{\mathrm{Mito,bulk}}}{vol_{\mathrm{myo,bulk}}} + J_{\mathrm{detach}} + \frac{D_{\mathrm{ATP}} SA_{\mathrm{myo,bulk\text{-}junc}}}{vol_{\mathrm{myo,bulk}} \Delta d_{\mathrm{diff}}} ([\mathrm{ADP}]_{\mathrm{myo,junc}} - [\mathrm{ADP}]_{\mathrm{myo,bulk}})$
$\frac{d[\mathrm{Ca}^{2+}]_{\mathrm{Mito,bulk}}}{dt}$	$b_{\mathrm{M}} \left(J_{\mathrm{MCU,bulk}} - J_{\mathrm{NCX,Mito,bulk}} + J_{\mathrm{mPTP,bulk}} - J_{\mathrm{Ca^{2+}\text{-}phos,Mito,bulk}} \right)$
$\frac{d[\mathrm{Pi}]_{\mathrm{Mito,bulk}}}{dt}$	$J_{\mathrm{PiC,bulk}} - J_{\mathrm{Ca^{2+}\text{-}phos,Mito,bulk}}$
$\frac{d[\mathrm{Ca}^{2+}\text{-}\mathrm{Pi}]_{\mathrm{Mito,bulk}}}{dt}$	$J_{\mathrm{Ca^{2+}\text{-}phos,Mito,bulk}}$
$\frac{d[\mathrm{ATP}]_{\mathrm{Mito,bulk}}}{dt}$	$J_{\mathrm{F1FO,bulk}} - J_{\mathrm{ANT,bulk}}$
$\frac{d[\mathrm{NADH}]_{\mathrm{Mito,bulk}}}{dt}$	$J_{\mathrm{PDH,bulk}} + J_{\mathrm{AGC,bulk}} - J_{\mathrm{Ox,bulk}}$
$\frac{dV_{\mathrm{Mito,bulk}}}{dt}$	$(a_1 J_{\mathrm{Ox,bulk}} - a_2 J_{\mathrm{F1FO,bulk}} - J_{\mathrm{Hleak,Mito,bulk}} - J_{\mathrm{NCX,Mito,bulk}} - 2J_{\mathrm{MCU,bulk}} - 2J_{\mathrm{mPTP,bulk}} - J_{\mathrm{ANT,bulk}} - J_{\mathrm{AGC,bulk}})/C_{\mathrm{Eff,Mito}}$

bioRxiv preprint doi: https://doi.org/10.1101/2025.05.22.655415; this version posted July 24, 2026. The copyright holder for this preprint (which was not certified by peer review) is the author/funder, who has granted bioRxiv a license to display the preprint in perpetuity. It is made available under a CC-BY 4.0 International license.

Table A7: Model parameters. Acronyms used here and not previously defined – AGC: aspartate/glutamate carrier, MM: Michaelis-Menten, KC: Krebs cycle, ETC: electron transport chain, MAS: malate-aspartate shuttle.

Index	Name	Description	Value	Reference
**Free parameters**				
1	A_a	Steepness factor for A	1.500×10^2 mV	(Wallinga et al., 1999)
			5.017×10^2 mV	Calibrated
2	$A_{h_{K\infty}}$	Steepness factor for h_K	7.500 mV	(Adrian et al., 1970)
			13.65 mV	Calibrated
3	$A_{S\infty}$	Steepness factor for S	5.800 mV	(Wallinga et al., 1999)
			4.205 mV	Calibrated
4	α_{h0}	Rate constant for h	$8.100\,s^{-1}$	(Wallinga et al., 1999)
			$16.2\,s^{-1}$	Calibrated
5	α_{m0}	Rate constant for m	$2.880 \times 10^2\,s^{-1}\,mV^{-1}$	(Wallinga et al., 1999)
			$1.402 \times 10^2\,s^{-1}\,mV^{-1}$	Calibrated
6	α_{n0}	Rate constant for n	$13.10\,s^{-1}\,mV^{-1}$	(Wallinga et al., 1999)
			$6.55\,s^{-1}\,mV^{-1}$	Calibrated
7	α_w	Rate constant for w_{RyR}	$1 \times 10^2\,s^{-1}$	(Standen and Stanfield, 1978)
			$1.998 \times 10^2\,s^{-1}$	Calibrated
8	β_{h0}	Rate constant for h	$4.380 \times 10^3\,s^{-1}$	(Wallinga et al., 1999)
			$4.10 \times 10^3\,s^{-1}$	Calibrated
9	β_{m0}	Rate constant for m	$1.380 \times 10^3\,s^{-1}$	(Wallinga et al., 1999)
			$4.051 \times 10^3\,s^{-1}$	Calibrated
10	β_{n0}	Rate constant for n	$67.00\,s^{-1}$	(Wallinga et al., 1999)
			$33.61\,s^{-1}$	Calibrated
11	C_{PM}	Specific membrane capacitance	$1.000 \times 10^{-5}\,pC\,mV^{-1}\,\mu m^{-2}$	(Cannon et al., 1993)
			$2.51 \times 10^{-5}\,pC\,mV^{-1}\,\mu m^{-2}$	Calibrated
12	K_{STIM1}	STIM1 Ca^{2+} sensitivity	$0.2500[Ca^{2+}]_{SR,\infty}$	(Luik et al., 2008)
			$0.2500[Ca^{2+}]_{SR,\infty}$	Varied*
13	I_{SL}	Amplitude of stimulus current	$1.989 \times 10^{-1}\,pA\,\mu m^{-2}$	(Wallinga et al., 1999)**
			$7.769 \times 10^{-1}\,pA\,\mu m^{-2}$	Calibrated

Continued on next page

bioRxiv preprint doi: https://doi.org/10.1101/2025.05.22.655415; this version posted July 24, 2026. The copyright holder for this preprint (which was not certified by peer review) is the author/funder, who has granted bioRxiv a license to display the preprint in perpetuity. It is made available under a CC-BY 4.0 International license.

Table A7: (continued.) Model parameters.

Index	Name	Description	Value	Reference
14	δ	Electrical distance	0.4000	(Standen and Stanfield, 1978)
			0.4448	Calibrated
15	f_{RyR}	Allosteric factor for RyR-DHPR coupling	0.2000	(Ríos et al., 1993)
			0.100	Calibrated
16	$g_{\mathrm{Cl^-}}$	$\mathrm{Cl^-}$ conductance	$0.1000\,\mathrm{pA\,mV^{-1}\,\mu m^{-2}}$	(Wallinga et al., 1999)**
			$0.0730\,\mathrm{pA\,mV^{-1}\,\mu m^{-2}}$	Calibrated
17	g_{K}	KDR conductance	$0.6480\,\mathrm{pA\,mV^{-1}\,\mu m^{-2}}$	(Wallinga et al., 1999)
			$0.3250\,\mathrm{pA\,mV^{-1}\,\mu m^{-2}}$	Calibrated
18	G_{K}	KIR conductance	$0.1110\,\mathrm{pA\,mV^{-1}\,\mu m^{-2}}$	(Wallinga et al., 1999)
			$0.3328\,\mathrm{pA\,mV^{-1}\,\mu m^{-2}}$	Calibrated
19	g_{Na}	$\mathrm{Na^+}$ conductance	$8.040\,\mathrm{pA\,mV^{-1}\,\mu m^{-2}}$	(Wallinga et al., 1999)
			$6.755\,\mathrm{pA\,mV^{-1}\,\mu m^{-2}}$	Calibrated
20	g_{NCX}	NCX conductance	$0.1290\,\mathrm{pA\,\mu m^{-2}}$	(Grandi et al., 2010)**
			$0.0645\,\mathrm{pA\,\mu m^{-2}}$	Calibrated
21	J_{NaK}	$\mathrm{Na^+}$-$\mathrm{K^+}$ pump conductance	$3.542\times10^{-5}\,\mathrm{pmol\,\mu m^{-2}\,s^{-1}}$	(Wallinga et al., 1999)**
			$5.608\times10^{-5}\,\mathrm{pmol\,\mu m^{-2}\,s^{-1}}$	Calibrated
22	K_{α_h}	Steepness factor for h	14.70 mV	(Cannon et al., 1993)
			14.59 mV	Calibrated
23	K_{α_m}	Steepness factor for m	10.00 mV	(Cannon et al., 1993)
			31.86 mV	Calibrated
24	K_{α_n}	Steepness factor for n	7.000 mV	(Cannon et al., 1993)
			1.755 mV	Calibrated
25	K_{β_h}	Slope factor for h	9.000 mV	(Cannon et al., 1993)
			35.10 mV	Calibrated
26	K_{β_m}	Slope factor for m	18.00 mV	(Cannon et al., 1993)
			40.79 mV	Calibrated
27	K_{β_n}	Slope factor for n	40.00 mV	(Cannon et al., 1993)
			79.96 mV	Calibrated

Continued on next page

bioRxiv preprint doi: https://doi.org/10.1101/2025.05.22.655415; this version posted July 24, 2026. The copyright holder for this preprint (which was not certified by peer review) is the author/funder, who has granted bioRxiv a license to display the preprint in perpetuity. It is made available under a CC-BY 4.0 International license.

Table A7: (continued.) Model parameters.

Index	Name	Description	Value	Reference
28	K_K	Dissociation constant for K^+	9.500×10^8 µM^2	(Standen and Stanfield, 1978)
			4.420×10^8 µM^2	Calibrated
29	K_{mK}	Affinity constant of the Na^+-K^+ pump for K^+	1.000×10^3 µM	(Wallinga et al., 1999)
			1.999×10^3 µM	Calibrated
30	K_{mNa}	Affinity constant of the Na^+-K^+ pump for Na^+	1.300×10^4 µM	(Wallinga et al., 1999)
			1.253×10^4 µM	Calibrated
31	K_{PMCA}	Michaelis constant	0.17 µM	(Kapela et al., 2008)
			0.1593 µM	Calibrated
32	K_{RyR}	Association rate constant for RyR	4.500 mV	(Ríos et al., 1993)
			3.358 mV	Calibrated
33	K_S	Dissociation constant for blocking S	1.000×10^6 µM^2	(Standen and Stanfield, 1978)
			1.551×10^6 µM^2	Calibrated
34	K_{SERCA}	SERCA Michaelis constant	1.000 µM	(Baylor and Hollingworth, 1998)
			1.999 µM	Calibrated
35	$K_{w_{r0}}$	Ca^{2+} sensitivity of w	1.000 µM	(Standen and Stanfield, 1978)
			1.859 µM	Calibrated
36	K_{dAct}	NCX Ca^{2+} dissociation constant	0.0500 µM	(Grandi et al., 2010)
			0.1 µM	Calibrated
37	$K_{Ca,i}$	NCX Ca^{2+} Michaelis constant	6.590 µM	(Grandi et al., 2010)
			10.06 µM	Calibrated
38	k_{sat}	NCX constant	0.3200	(Grandi et al., 2010)
			0.64	Calibrated
39	ν_{NCX}	NCX constant	0.2700	(Grandi et al., 2010)
			0.5390	Calibrated
40	S_i	Intracellular blocking concentration	1×10^4 µM	(Standen and Stanfield, 1978)
			3.336×10^4 µM	Calibrated
41	τ_{SOCE}	Activation time for SOCE	0.0100 s	This study
			0.020 s	Calibrated

Continued on next page

bioRxiv preprint doi: https://doi.org/10.1101/2025.05.22.655415; this version posted July 24, 2026. The copyright holder for this preprint (which was not certified by peer review) is the author/funder, who has granted bioRxiv a license to display the preprint in perpetuity. It is made available under a CC-BY 4.0 International license.

Table A7: (continued.) Model parameters.

Index	Name	Description	Value	Reference
42	ν_{SERCA}	Maximum SERCA uptake rate	1.134×10^4 µm µM s^{-1}	(Shorten et al., 2007)***
			5.670×10^3 µm µM s^{-1}	Calibrated
43	$J_{\text{PMCA},0}$	Max PMCA flux	39.75 µM s^{-1}	(Kapela et al., 2008)***
			20.00 µM s^{-1}	Calibrated
44	$\nu_{\text{leak,SR}}$	SR leak permeability	0.4652 µm s^{-1}	This study
			0.2326 µm s^{-1}	Calibrated
46	$k_{\text{on,CA}}$	Ca^{2+}-ATP binding rate	15.00 µM^{-1} s^{-1}	(Baylor and Hollingworth, 1998)**
			7.500 µM^{-1} s^{-1}	Calibrated
47	$k_{\text{off,CA}}$	Ca^{2+}-ATP unbinding rate	3.000×10^2 s^{-1}	(Baylor and Hollingworth, 1998)**
			6.0×10^2 s^{-1}	Calibrated
48	$k_{\text{on,CP}}$	Ca^{2+}-parvalbumin binding rate	41.70 µM^{-1} s^{-1}	(Baylor and Hollingworth, 1998)
			20.86 µM^{-1} s^{-1}	Calibrated
49	$k_{\text{off,CP}}$	Ca^{2+}-parvalbumin unbinding rate	0.5000 s^{-1}	(Baylor and Hollingworth, 1998)
			0.2658 s^{-1}	Calibrated
50	$k_{\text{on,MP}}$	Mg^{2+}-parvalbumin binding rate	0.0330 µM^{-1} s^{-1}	(Baylor and Hollingworth, 1998)
			0.0660 µM^{-1} s^{-1}	Calibrated
51	$k_{\text{off,MP}}$	Mg^{2+}-parvalbumin unbinding rate	3.000 s^{-1}	(Baylor and Hollingworth, 1998)
			5.515 s^{-1}	Calibrated
52	$[\text{Parv}]_{\text{tot}}$	Total parvalbumin concentration	1.500×10^3 µM	(Baylor and Hollingworth, 1998)
			7.506×10^2 µM	Calibrated
53	$k_{\text{on,MA}}$	$[Mg^{2+}]$-ATP binding rate	1.500 µM s^{-1}	(Baylor and Hollingworth, 1998)
			2.960 µM^{-1} s^{-1}	Calibrated
54	$k_{\text{off,MA}}$	$[Mg^{2+}]$-ATP unbinding rate	150.0 s^{-1}	(Baylor and Hollingworth, 1998)
			76.10 s^{-1}	Calibrated

Continued on next page

bioRxiv preprint doi: https://doi.org/10.1101/2025.05.22.655415; this version posted July 24, 2026. The copyright holder for this preprint (which was not certified by peer review) is the author/funder, who has granted bioRxiv a license to display the preprint in perpetuity. It is made available under a CC-BY 4.0 International license.

Table A7: (continued.) Model parameters.

Index	Name	Description	Value	Reference
55	k_{hyd}	Maximal rate of ATP hydrolysis	$100.0\,\mu\mathrm{M\,s^{-1}}$	(Wacquier et al., 2016)
			$68.95\,\mu\mathrm{M^{-1}\,s^{-1}}$	Calibrated
56	k_{H}	Michaelis constant for ATP hydrolysis	$1.000\times10^{3}\,\mu\mathrm{M}$	(Wacquier et al., 2016)
			$5.00\times10^{2}\,\mu\mathrm{M}$	Calibrated
57	k_{prod}	ATP production rate	$100\,\mathrm{s^{-1}}$	This study
			$51.44\,\mathrm{s^{-1}}$	Calibrated
58	$[Mg^{2+}]$	Myoplasmic $[Mg^{2+}]$	$1.000\times10^{3}\,\mu\mathrm{M}$	(Baylor and Hollingworth, 1998)
			$2.00\times10^{3}\,\mu\mathrm{M}$	Calibrated
59	$k_{\mathrm{on,T1}}$	Ca^{2+}-troponin binding rate	$177.0\,\mu\mathrm{M^{-1}\,s^{-1}}$	(Baylor and Hollingworth, 2007)
			$88.50\,\mu\mathrm{M^{-1}\,s^{-1}}$	Calibrated
60	$k_{\mathrm{off,T1}}$	Ca^{2+}-troponin unbinding rate	$1.544\times10^{3}\,\mathrm{s^{-1}}$	(Baylor and Hollingworth, 2007)
			$3.086\times10^{3}\,\mathrm{s^{-1}}$	Calibrated
61	k_{unblock}	Ca^{2+}-RU activation rate	$150.0\,\mathrm{s^{-1}}$	(Razumova et al., 2000)***
			$75.06\,\mathrm{s^{-1}}$	Calibrated
62	k_{block}	Ca^{2+}-RU deactivation rate	$50.00\,\mathrm{s^{-1}}$	(Razumova et al., 2000)
			$99.98\,\mathrm{s^{-1}}$	Calibrated
63	f_0	Rate of crossbridge attachment	$1.500\times10^{3}\,\mathrm{s^{-1}}$	(Shorten et al., 2007)
			$7.822\times10^{2}\,\mathrm{s^{-1}}$	Calibrated
64	f_{p}	Rate of crossbridge detachment	$1.500\times10^{4}\,\mathrm{s^{-1}}$	(Shorten et al., 2007)
			$2.999\times10^{4}\,\mathrm{s^{-1}}$	Calibrated
65	h_0	Forward rate of the power stroke	$240\,\mathrm{s^{-1}}$	(Shorten et al., 2007)
			$120.1\,\mathrm{s^{-1}}$	Calibrated
66	h_{p}	Backward rate of the power stroke	$0.0600\,\mu\mathrm{M^{-1}\,s^{-1}}$	(Senneff and Lowery, 2021)
			$0.0709\,\mu\mathrm{M^{-1}\,s^{-1}}$	Calibrated
67	g_0	Rate of myosin head unbinding	$0.1714\,\mu\mathrm{M^{-1}\,s^{-1}}$	(Senneff and Lowery, 2021)
			$0.3428\,\mu\mathrm{M^{-1}\,s^{-1}}$	Calibrated

Continued on next page

bioRxiv preprint doi: https://doi.org/10.1101/2025.05.22.655415; this version posted July 24, 2026. The copyright holder for this preprint (which was not certified by peer review) is the author/funder, who has granted bioRxiv a license to display the preprint in perpetuity. It is made available under a CC-BY 4.0 International license.

Table A7: (continued.) Model parameters.

Index	Name	Description	Value	Reference
68	PP	Phosphate solubility product	6.000×10^{6} µM^2	(Allen and Westerblad, 2001)
			1.117×10^{7} µM^2	Calibrated
69	k_p	Rate of phosphate transport into the SR	8.421×10^{-3} µm s^{-1}	(Shorten et al., 2007)***
			1.625×10^{-2} µm s^{-1}	Calibrated
70	A_p	Rate of phosphate precipitation from the SR	1.000×10^{-6} µM^{-3} s^{-1}	(Shorten et al., 2007)
			2.00×10^{-6} µM^{-3} s^{-1}	Calibrated
71	B_p	Rate of phosphate precipitate solubilization	1.000×10^{-7} µM^{-2} s^{-1}	(Shorten et al., 2007)
			1.995×10^{-7} µM^{-2} s^{-1}	Calibrated
73	$[\mathrm{Trop}]_\mathrm{tot}$	Total troponin concentration	140 µM	(Baylor and Hollingworth, 1998)
			70.05 µM	Calibrated
74	$[\mathrm{Pi}]_\mathrm{SR,init}$	Initial SR phosphate concentration	3.000×10^{3} µM	This study
			1.504×10^{3} µM	Calibrated
75	$[\mathrm{Pi}]_\mathrm{myo,init}$	Initial myoplasmic phosphate concentration	3.000×10^{3} µM	This study
			2.635×10^{3} µM	Calibrated
76	V_a	Half-maximal voltage for A	70.00 mV	(Wallinga et al., 1999)
			80.00 mV	Calibrated
77	V_h	Half-maximal voltage for h	−45.00 mV	(Cannon et al., 1993)
			−47.99 mV	Calibrated
78	$V_{h_{\mathrm{K}\infty}}$	Half-maximal voltage for h_K	−40.00 mV	(Adrian et al., 1970)
			−30.22 mV	Calibrated
79	V_m	Half-maximal voltage for m	−46.00 mV	(Cannon et al., 1993)
			−45.40 mV	Calibrated
80	V_n	Half-maximal voltage for n	−40.00 mV	(Cannon et al., 1993)
			−20.00 mV	Calibrated
81	$V_{S\infty}$	Half-maximal voltage for S	−78.00 mV	(Wallinga et al., 1999)
			−81.38 mV	Calibrated
82	V_τ	Voltage parameter for τ_S	90.00 mV	(Ruff and Whittlesey, 1992)
			83.12 mV	Calibrated
83	V_RyR	RyR Activation rate constant	−20.00 mV	(Ríos et al., 1993)

Continued on next page

bioRxiv preprint doi: https://doi.org/10.1101/2025.05.22.655415; this version posted July 24, 2026. The copyright holder for this preprint (which was not certified by peer review) is the author/funder, who has granted bioRxiv a license to display the preprint in perpetuity. It is made available under a CC-BY 4.0 International license.

Table A7: (continued.) Model parameters.

Index	Name	Description	Value	Reference
			$-30.00\,\mathrm{mV}$	Calibrated
84	k_{ATP}	ATP dependence of SR Ca pump	$0.040\,00\,\mathrm{\mu M}$	(Lytton et al., 1992)
			$0.079\,78\,\mathrm{\mu M}$	Calibrated
85	$K_{\mathrm{Na,i}}$	Myoplasmic Na^+ Michaelis constant	$1.229 \times 10^4\,\mathrm{\mu M}$	(Grandi et al., 2010)
			$2.400 \times 10^4\,\mathrm{\mu M}$	Calibrated
86	$K_{\mathrm{Na,o}}$	Extracellular Na^+ Michaelis constant	$8.750 \times 10^4\,\mathrm{\mu M}$	(Grandi et al., 2010)
			$1.739 \times 10^5\,\mathrm{\mu M}$	Calibrated
87	$K_{\mathrm{Ca,o}}$	Extracellular Ca^{2+} Michaelis constant	$1.300 \times 10^3\,\mathrm{\mu M}$	(Grandi et al., 2010)
			$1.477 \times 10^3\,\mathrm{\mu M}$	Calibrated
89	$g_{\mathrm{leak,SL}}$	Ca^{2+} leak conductance	$1 \times 10^{-5}\,\mathrm{pA\,mV^{-1}\,\mu m^{-2}}$	This study
			$1.999 \times 10^{-5}\,\mathrm{pA\,mV^{-1}\,\mu m^{-2}}$	Calibrated
90	L_{RyR}	Baseline release of Ca^{2+}	5×10^5	(Ríos et al., 1993)
			6.226×10^5	Calibrated
92	$\nu_{\mathrm{max,RyR}}$	Maximum release rate of Ca^{2+} from RyR	$1.02 \times 10^8\,\mathrm{\mu m\,s^{-1}}$	(Ríos et al., 1993)†
			$2.022 \times 10^8\,\mathrm{\mu m\,s^{-1}}$	Calibrated
93	$k_{\mathrm{on,T2}}$	RU activation rate	$88.5\,\mathrm{\mu M^{-1}\,s^{-1}}$	(Baylor and Hollingworth, 2007)
			$44.25\,\mathrm{\mu M^{-1}\,s^{-1}}$	Calibrated
94	$k_{\mathrm{off,T2}}$	RU deactivation rate	$17.1\,\mathrm{s^{-1}}$	(Baylor and Hollingworth, 2007)
			$34.15\,\mathrm{s^{-1}}$	Calibrated
95	g_{Orai1}	SOCE conductance	$4.752 \times 10^{-5}\,\mathrm{pA\,mV^{-1}\,\mu m^{-2}}$	(Luik et al., 2008)††
			$2.393 \times 10^{-5}\,\mathrm{pA\,mV^{-1}\,\mu m^{-2}}$	Calibrated
96	$[\mathrm{CSQN}]_{\mathrm{tot}}$	Total Calsequestrin concentration	$3.100 \times 10^4\,\mathrm{\mu M}$	(Cannell and Allen, 1984)
			$6.200 \times 10^4\,\mathrm{\mu M}$	Calibrated
97	K_{CSQN}	CSQN-Ca^{2+} dissociation constant	$800\,\mathrm{\mu M}$	(Ostwald and MacLennan, 1974)
			$400.0\,\mathrm{\mu M}$	Calibrated
98	$k_{\mathrm{off,CSQN}}$	CSQN-Ca^{2+} unbinding rate in SR	$5\,\mathrm{s^{-1}}$	(Cannell and Allen, 1984)
			$9.999\,\mathrm{s^{-1}}$	Calibrated
99	d_{diff}	characteristic diffusion length	$0.1\,\mathrm{\mu m}$	This study
			$0.1999\,\mathrm{\mu m^2\,s^{-1}}$	Calibrated
100	D_{ion}	Ion diffusion coefficient	$1000\,\mathrm{\mu m}$	This study†††
			$1062\,\mathrm{\mu m^2\,s^{-1}}$	Calibrated

Continued on next page

bioRxiv preprint doi: https://doi.org/10.1101/2025.05.22.655415; this version posted July 24, 2026. The copyright holder for this preprint (which was not certified by peer review) is the author/funder, who has granted bioRxiv a license to display the preprint in perpetuity. It is made available under a CC-BY 4.0 International license.

Table A7: (continued.) Model parameters.

Index	Name	Description	Value	Reference
101	D_{ATP}	ATP diffusion coefficient	$500\,\mu\mathrm{m}^2\,\mathrm{s}^{-1}$	This study[†††]
			$992.6\,\mu\mathrm{m}^2\,\mathrm{s}^{-1}$	Calibrated
102	D_{Parv}	Parv diffusion coefficient	$1\,\mu\mathrm{m}^2\,\mathrm{s}^{-1}$	This study[†††]
			$0.5120\,\mu\mathrm{m}^2\,\mathrm{s}^{-1}$	Calibrated
103	D_{CSQN}	CSQN diffusion coefficient	$0.1\,\mu\mathrm{m}^2\,\mathrm{s}^{-1}$	This study[†††]
			$0.1769\,\mu\mathrm{m}^2\,\mathrm{s}^{-1}$	Calibrated
104	D_{Pi}	Phosphate diffusion coefficient	$500\,\mu\mathrm{m}^2\,\mathrm{s}^{-1}$	This study[†††]
			$640.7\,\mu\mathrm{m}^2\,\mathrm{s}^{-1}$	Calibrated
105	R_a	Access resistance for current from SL to TT	$1.500 \times 10^{-2}\,\mu\mathrm{m}^2\,\mathrm{mV}\,\mathrm{pA}^{-1}$	(Cannon et al., 1993)
			$3.00 \times 10^{-2}\,\mu\mathrm{m}^2\,\mathrm{mV}\,\mathrm{pA}^{-1}$	Calibrated
106	$[\mathrm{ATP}]_{\mathrm{set}}$	Myoplasmic ATP set-point	$700\,\mu\mathrm{M}$	This study
			$350.5\,\mu\mathrm{M}$	Calibrated
**Mitochondria-related parameters (separate estimation)**				
M1	K_{db}	Ca^{2+}-phosphate dissociation constant	$5 \times 10^2\,\mu\mathrm{M}$	This study[§]
			$2.5 \times 10^2\,\mu\mathrm{M}$	Calibrated
M2	b_{M}	Rapid-buffering free Ca^{2+} fraction	0.01	This study[§]
			0.005	Calibrated
M3	ν_{MCU}	MCU rate constant	$6.0 \times 10^{-4}\,\mu\mathrm{M}\,\mathrm{s}^{-1}$	(Wacquier et al., 2016)
			$1.20 \times 10^{-3}\,\mu\mathrm{M}\,\mathrm{s}^{-1}$	Calibrated
M4	K_{trans}	Dissoc. constant for Ca^{2+} translocation by MCU	$6.0\,\mu\mathrm{M}$	(Wacquier et al., 2016)
			$3.0\,\mu\mathrm{M}$	Calibrated
M5	K_{act}	Dissoc. constant for Ca^{2+}-mediated MCU activation	$0.38\,\mu\mathrm{M}$	(Magnus and Keizer, 1997)
			$0.1903\,\mu\mathrm{M}$	Calibrated
M6	L_{MCU}	Allosteric equilibrium constant for MCU	50	(Magnus and Keizer, 1997)
			37.42	Calibrated
M7	p_1	Voltage dependence coefficient for MCU	$0.1\,\mathrm{mV}^{-1}$	(Wacquier et al., 2016)
			0.1302	Calibrated
M8	$V_{\mathrm{NCX,Mito}}$	Mitochondrial NCX rate constant	$0.35\,\mu\mathrm{M}\,\mathrm{s}^{-1}$	(Wacquier et al., 2016)
			$0.1751\,\mu\mathrm{M}\,\mathrm{s}^{-1}$	Calibrated
M9	p_2	Mito NCX voltage dependence coefficient	$1.6 \times 10^{-2}\,\mathrm{mV}^{-1}$	(Wacquier et al., 2016)
			$8.0 \times 10^{-3}\,\mathrm{mV}^{-1}$	Calibrated
M10	k_{mPTP}	Rate constant for mPTP	$8.0 \times 10^{-3}\,\mathrm{s}^{-1}$	(Wacquier et al., 2016)
			$4.004 \times 10^{-3}\,\mathrm{s}^{-1}$	Calibrated
M11	p_3	Voltage dependence coefficient for mPTP	$5.0 \times 10^{-2}\,\mathrm{mV}^{-1}$	(Wacquier et al., 2016)
			$9.999 \times 10^{-2}\,\mathrm{mV}^{-1}$	Calibrated

Continued on next page

bioRxiv preprint doi: https://doi.org/10.1101/2025.05.22.655415; this version posted July 24, 2026. The copyright holder for this preprint (which was not certified by peer review) is the author/funder, who has granted bioRxiv a license to display the preprint in perpetuity. It is made available under a CC-BY 4.0 International license.

Table A7: (continued.) Model parameters.

Index	Name	Description	Value	Reference
M12	k_{Gly}	Glycolysis rate	$4.5 \times 10^{2}\,\mathrm{s^{-1}}$	(Bertram et al., 2006)*
			$2.257 \times 10^{2}\,\mathrm{s^{-1}}$	Calibrated
M13	K_{MCa}	MM constant for Ca^{2+}-dependent KC activation	$0.1\,\mathrm{\mu M}$	(Wacquier et al., 2016)
			$0.0503\,\mathrm{\mu M}$	Calibrated
M14	K_{mNAD}	MM constant for NAD^{+} consumption by the KC	1.0	(Bertram et al., 2006)
			1.996	Calibrated
M15	ν_{Ox}	NADH oxidation rate constant (ETC)	$6.0 \times 10^{2}\,\mathrm{\mu M\,s^{-1}}$	(Bertram et al., 2006)
			$1.2 \times 10^{3}\,\mathrm{\mu M\,s^{-1}}$	Calibrated
M16	K_{Ox}	MM constant for NADH oxidation (ETC)	$1.0 \times 10^{2}\,\mathrm{\mu M}$	(Bertram et al., 2006)
			$1.844 \times 10^{2}\,\mathrm{\mu M}$	Calibrated
M17	p_4	Voltage-dependence coefficient for ETC (shift)	$1.77 \times 10^{2}\,\mathrm{mV}$	(Bertram et al., 2006)
			$1.829 \times 10^{2}\,\mathrm{mV}$	Calibrated
M18	p_5	Voltage-dependence coefficient for ETC (sensitivity)	$5.0\,\mathrm{mV}$	(Bertram et al., 2006)
			$2.5\,\mathrm{mV}$	Calibrated
M19	ν_{AGC}	Rate constant for NADH production via MAS	$25.0\,\mathrm{\mu M\,s^{-1}}$	(Wacquier et al., 2016)
			$12.51\,\mathrm{\mu M\,s^{-1}}$	Calibrated
M20	K_{AGC}	Dissociation constant of Ca^{2+} from AGC	$0.14\,\mathrm{\mu M}$	(Contreras et al., 2007)
			$0.28\,\mathrm{\mu M}$	Calibrated
M21	K_{iCa}	Constant for inhibition of AGC by cytosolic Ca^{2+}	$0.1\,\mathrm{\mu M}$	(Wacquier et al., 2016)
			$0.1978\,\mathrm{\mu M}$	Calibrated
M22	ν_{F1FO}	Rate constant for F1FO ATPase	$3.5 \times 10^{4}\,\mathrm{\mu M\,s^{-1}}$	(Bertram et al., 2006)
			$1.750 \times 10^{4}\,\mathrm{\mu M\,s^{-1}}$	Calibrated
M23	p_6	Voltage dependence coefficient for ATPase (shift)	$1.9 \times 10^{2}\,\mathrm{mV}$	(Bertram et al., 2006)
			$2.0 \times 10^{2}\,\mathrm{mV}$	Calibrated
M24	p_7	Voltage dependence coefficient for ATPase (sensitivity)	$8.5\,\mathrm{mV}$	(Bertram et al., 2006)
			$4.25\,\mathrm{mV}$	Calibrated
M25	K_{iATP}	Constant for inhibition of ATPase activity by ATP	$1.0 \times 10^{4}\,\mathrm{mV}$	(Bertram et al., 2006)
			$5.0 \times 10^{3}\,\mathrm{mV}$	Calibrated
M26	ν_{ANT}	Rate constant for ANTs	$5.0 \times 10^{3}\,\mathrm{\mu M\,s^{-1}}$	(Bertram et al., 2006)
			$2.5 \times 10^{3}\,\mathrm{\mu M\,s^{-1}}$	Calibrated

Continued on next page

bioRxiv preprint doi: https://doi.org/10.1101/2025.05.22.655415; this version posted July 24, 2026. The copyright holder for this preprint (which was not certified by peer review) is the author/funder, who has granted bioRxiv a license to display the preprint in perpetuity. It is made available under a CC-BY 4.0 International license.

Table A7: (continued.) Model parameters.

Index	Name	Description	Value	Reference
M27	α_c	Factor for cytosolic ADP and ATP buffering	0.111	(Fall and Keizer, 2001)
			0.2215	Calibrated
M28	α_{Mito}	Factor for mitochondrial ADP and ATP buffering	0.139	(Fall and Keizer, 2001)
			0.069 52	Calibrated
M29	f_{ANT}	Controls sensitivity of ANTs to voltage	0.5	(Wacquier et al., 2016)
			0.25	Calibrated
M30	p_8	Voltage-dependent proton leak parameter	$2.0\,\mu M\,s^{-1}\,mV^{-1}$	(Bertram et al., 2006)
			$4.0\,\mu M\,s^{-1}\,mV^{-1}$	Calibrated
M31	p_9	Voltage-independent proton leak parameter	$-30\,\mu M\,s^{-1}$	(Bertram et al., 2006)
			$-18.03\,\mu M\,s^{-1}$	Calibrated
M32	ν_{PiC}	Rate constant for PiC	$5.0 \times 10^3\,\mu M\,s^{-1}$	This study§§
			$2.5 \times 10^3\,\mu M\,s^{-1}$	Calibrated
M33	C_{pEff}	Effective mitochondrial membrane capacitance	$1.8\,\mu M\,mV^{-1}$	(Bertram et al., 2006)
			$3.6\,\mu M\,mV^{-1}$	Calibrated
M34	a_1	Contribution of NADH consumption to voltage change	20	(Wacquier et al., 2016)
			10	Calibrated
M35	a_2	Contribution of ATPase activity to voltage change	3.43	(Bertram et al., 2006)
			1.715	Calibrated
M36	$K_{Pi,i}$	Hill constant for PiC activity	$1 \times 10^3\,\mu M$	This study§§
			$5.001 \times 10^2\,\mu M$	Calibrated
M37	$k_{off,B}$	Ca^{2+}-phosphate unbinding rate in mitochondria	$1.0\,s^{-1}$	This study§
			$1.996\,s^{-1}$	Calibrated
M38	$[ANP]_{tot}$	Total mitochondrial conc. of adenine nucleotides	$1.5 \times 10^4\,\mu M$	(Bertram et al., 2006)
			$3.0 \times 10^4\,\mu M$	Calibrated
M39	$[NAD]_{tot}$	Total mitochondrial conc. of NAD and NADH	$2.5 \times 10^2\,\mu M$	(Wacquier et al., 2016)
			$4.997 \times 10^2\,\mu M$	Calibrated
M40	$K_{Pi,M}$	PiC Hill constant for mitochondrial phosphate	$1.0 \times 10^4\,\mu M$	(Wacquier et al., 2016)
			$5.224 \times 10^3\,\mu M$	Calibrated
**Fixed parameters**				
Temperature and extracellular ion concentrations are fixed except in cases tested for parameter estimation (see Figure 2 caption).				
	$[Ca^{2+}]_{EC}$	Bulk extracellular Ca^{2+} concentration	$1.3 \times 10^3\,\mu M$	(Senneff and Lowery, 2021)

Continued on next page

bioRxiv preprint doi: https://doi.org/10.1101/2025.05.22.655415; this version posted July 24, 2026. The copyright holder for this preprint (which was not certified by peer review) is the author/funder, who has granted bioRxiv a license to display the preprint in perpetuity. It is made available under a CC-BY 4.0 International license.

Table A7: (continued.) Model parameters.

Index	Name	Description	Value	Reference
	$[Cl^-]_{EC}$	Bulk extracellular Cl^- concentration	1.280×10^5 µM	(Senneff and Lowery, 2021)
	$[K^+]_{EC}$	Bulk extracellular K^+ concentration	4.000×10^3 µM	(Senneff and Lowery, 2021)
	$[Na^+]_{EC}$	Bulk extracellular Na^+ concentration	1.470×10^5 µM	(Senneff and Lowery, 2021)
	$N_{\text{ATP-per-NADH}}$	Ratio of ATP per NADH produced in glycolysis	1	
	$D_{\text{ion,EC}}$	Diffusion coefficient for extracellular ions	1.000×10^3 µm^2 s^{-1}	This study
	T	Temperature	295.15 K	
	F	Faraday's constant	9.6485×10^4 pC pmol^{-1}	
	N_A	Avogadro's number	6.022×10^{11} molecules pmol^{-1}	
	R	Gas constant	8.3145×10^3 fJ pmol^{-1} K^{-1}	
	$\eta_{\text{conv},1}$	Unit conversion factor 1	1×10^9 µM µm^3 pmol^{-1}	
	$\eta_{\text{conv},2}$	Unit conversion factor 2	602.2 molecules µm^{-3} µM^{-1}	
Temperature coefficients.		In all cases $\Delta T = T - 293.15$		
	Q_{NCX}		$1.57^{\Delta T/10}$	(Grandi et al., 2010)
	Q_{KDR}		$1.5^{\Delta T/10}$	(Standen and Stanfield, 1978)
	Q_{Na}		$1.5^{\Delta T/10}$	(Stephanova and Dimitrov, 1982)
	Q_{KIR}		$1.55^{\Delta T/10}$	(Caffier and Shvinka, 1986)
	Q_{NaK}		1	(Senneff and Lowery, 2021)
	Q_{Cl}		$1.5^{\Delta T/10}$	(Fortune and Lowery, 2009)
	$Q_{C_{\text{SL}}}$		$1.02^{\Delta T/10}$	(Fortune and Lowery, 2009)
	Q_{α_n}		$2.5^{\Delta T/10}$	(Standen and Stanfield, 1978)
	Q_{α_m}		$2.3^{\Delta T/10}$	(Fortune and Lowery, 2009)

Continued on next page

bioRxiv preprint doi: https://doi.org/10.1101/2025.05.22.655415; this version posted July 24, 2026. The copyright holder for this preprint (which was not certified by peer review) is the author/funder, who has granted bioRxiv a license to display the preprint in perpetuity. It is made available under a CC-BY 4.0 International license.

Table A7: (continued.) Model parameters.

Index	Name	Description	Value	Reference
	Q_{α_h}		$2.5^{\Delta T/10}$	(Standen and Stanfield, 1978)
	Q_{β_n}		$2.5^{\Delta T/10}$	(Standen and Stanfield, 1978)
	Q_{β_m}		$2.3^{\Delta T/10}$	(Fortune and Lowery, 2009)
	Q_{β_h}		$2.3^{\Delta T/10}$	(Standen and Stanfield, 1978)
	Q_{SERCA}		$2.6^{\Delta T/10}$	(Shannon et al., 2004)
	Q_{PMCA}		$2.35^{\Delta T/10}$	(Shannon et al., 2004)

*Fixed to 0.25 times the resting SR Ca^{2+} without SOCE in baseline simulations, varied in Figure 8 and Figures A7 and A8.

**Order-of-magnitude estimate from source studies.

***Flux magnitude corrected according to the ratio of volumes of cells (or volume of compartments) in the referenced studies vs. myofiber volume here.

†Assuming an SR Ca^{2+} concentration of 500 µM, corresponds to a total flux (by volume) of 150 µM/s, close to the measured values in (Ríos et al., 1993).

††Converted from the 1 A/F current density measured in (Luik et al., 2008), assuming specific capacitance of $1.0\,\mu\mathrm{F\,cm^{-2}}$ and $E_{\mathrm{Ca^{2+}}} - V_{\mathrm{rest}} \approx 200\,\mathrm{mV}$.

†††Order-of-magnitude estimate based on molecular size.

§ Initial estimates for phosphate-independent Ca^{2+} buffering and Ca^{2+}-phosphate dissociation coefficient informed by data from (Wei et al., 2015), given initial (phosphate-free) fraction of free Ca^{2+} and concentrations of phosphate tested. The Ca^{2+}-phosphate off-rate was fixed at $1.0\,\mathrm{s^{-1}}$ and did not affect the fit.

§§ The order of magnitude for maximum transport rate and Hill constant for PiC were converted from previous measurements (maximum rate of 100-1000 $\mathrm{nmol\,mg^{-1}\,min^{-1}}$ in terms of mitochondrial protein content and 50% maximum flux at 1-2 mM phosphate (Seifert et al., 2015; Wohlrab, 1980; Coty and Pedersen, 1974)), assuming total mitochondrial density of 1.2 g/mL (Glas and Bahr, 1966) and mitochondrial protein content of about 25% (Fuchs et al., 2020).

Appendix B: Supplementary figures

bioRxiv preprint doi: https://doi.org/10.1101/2025.05.22.655415; this version posted July 24, 2026. The copyright holder for this preprint (which was not certified by peer review) is the author/funder, who has granted bioRxiv a license to display the preprint in perpetuity. It is made available under a CC-BY 4.0 International license.

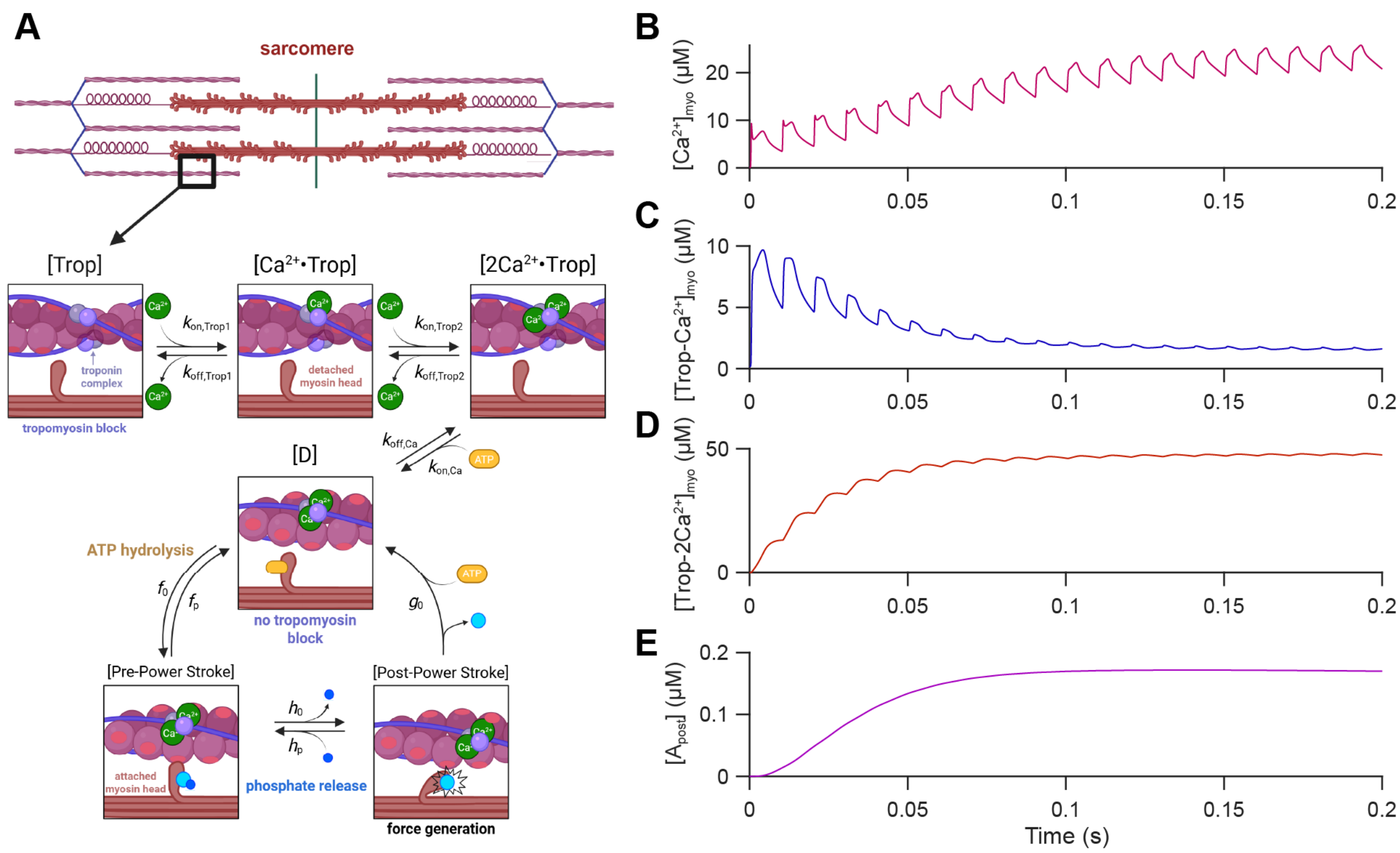

Figure A1: Summary of six-state cross-bridge model. A) Schematic of cross-bridge model. Two calcium ions bind to troponin, facilitating unblocking of the myosin-head binding sites on actin. ATP hydrolysis enables myosin-head binding to actin, and phosphate release drives the power stroke. Figure created in BioRender. B-E) Model predictions of myoplasmic Ca^{2+} (B) and different cross-bridge states during repetitive stimuli, including troponin bound to one Ca^{2+} (C), troponin bound to two Ca^{2+} (D), and cross-bridges post-power stroke (E).

bioRxiv preprint doi: https://doi.org/10.1101/2025.05.22.655415; this version posted July 24, 2026. The copyright holder for this preprint (which was not certified by peer review) is the author/funder, who has granted bioRxiv a license to display the preprint in perpetuity. It is made available under a CC-BY 4.0 International license.

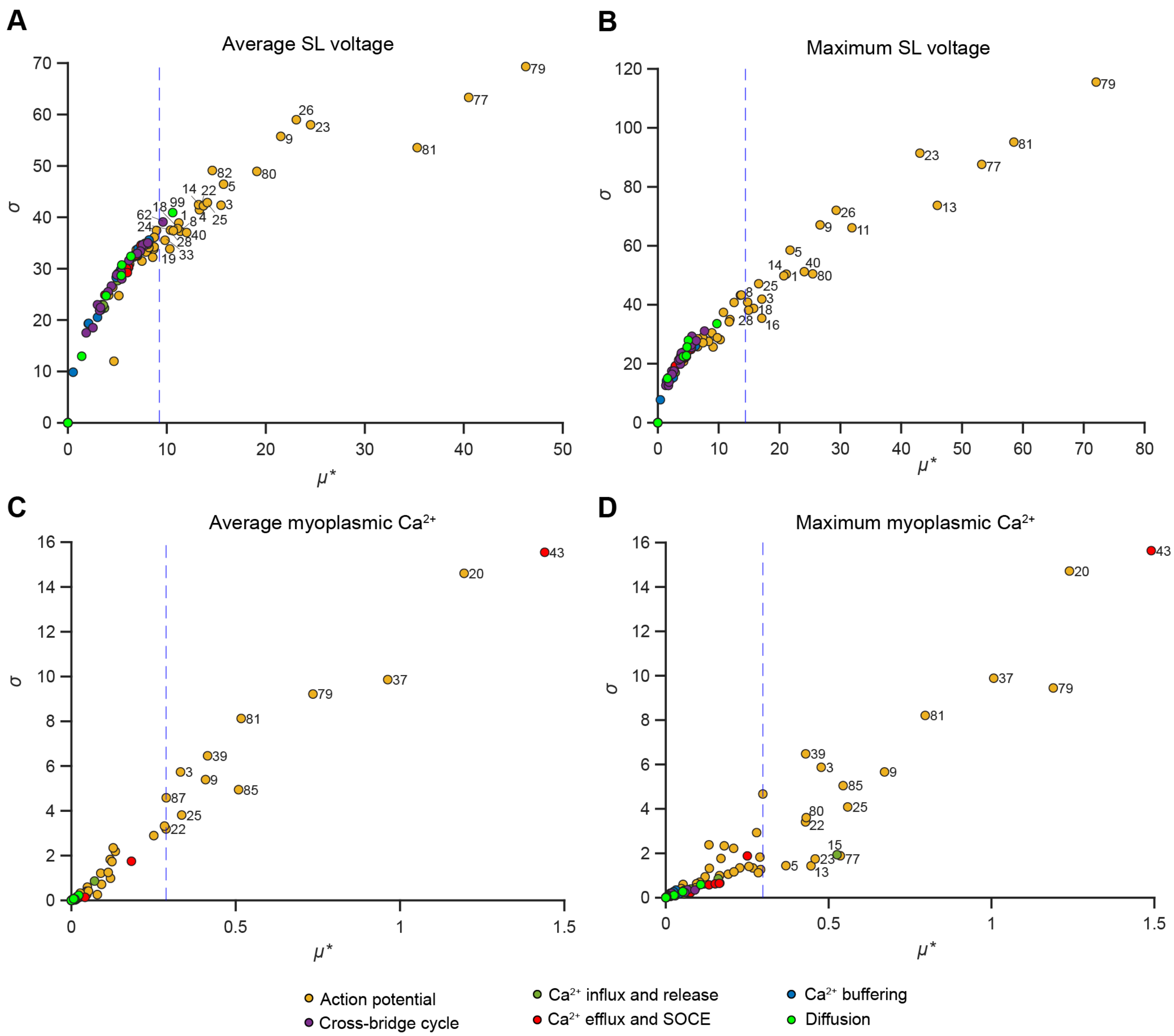

Figure A2: Morris sensitivity analysis for four main quantities of interest. Model sensivity was assessed with respect to average sarcolemma voltage (A), peak sarcolemma voltage (B), average myoplasmic Ca^{2+} (C), and peak myoplasmic Ca^{2+} (D). In plots associated with each QOI, the horizontal axis displays the absolute value of the mean, μ^* and the vertical axis shows the standard deviation, σ, of elementary effects. All parameters with μ^* greater than 20% of the maximum value are labeled according to their assigned number in Table A7.

bioRxiv preprint doi: https://doi.org/10.1101/2025.05.22.655415; this version posted July 24, 2026. The copyright holder for this preprint (which was not certified by peer review) is the author/funder, who has granted bioRxiv a license to display the preprint in perpetuity. It is made available under a CC-BY 4.0 International license.

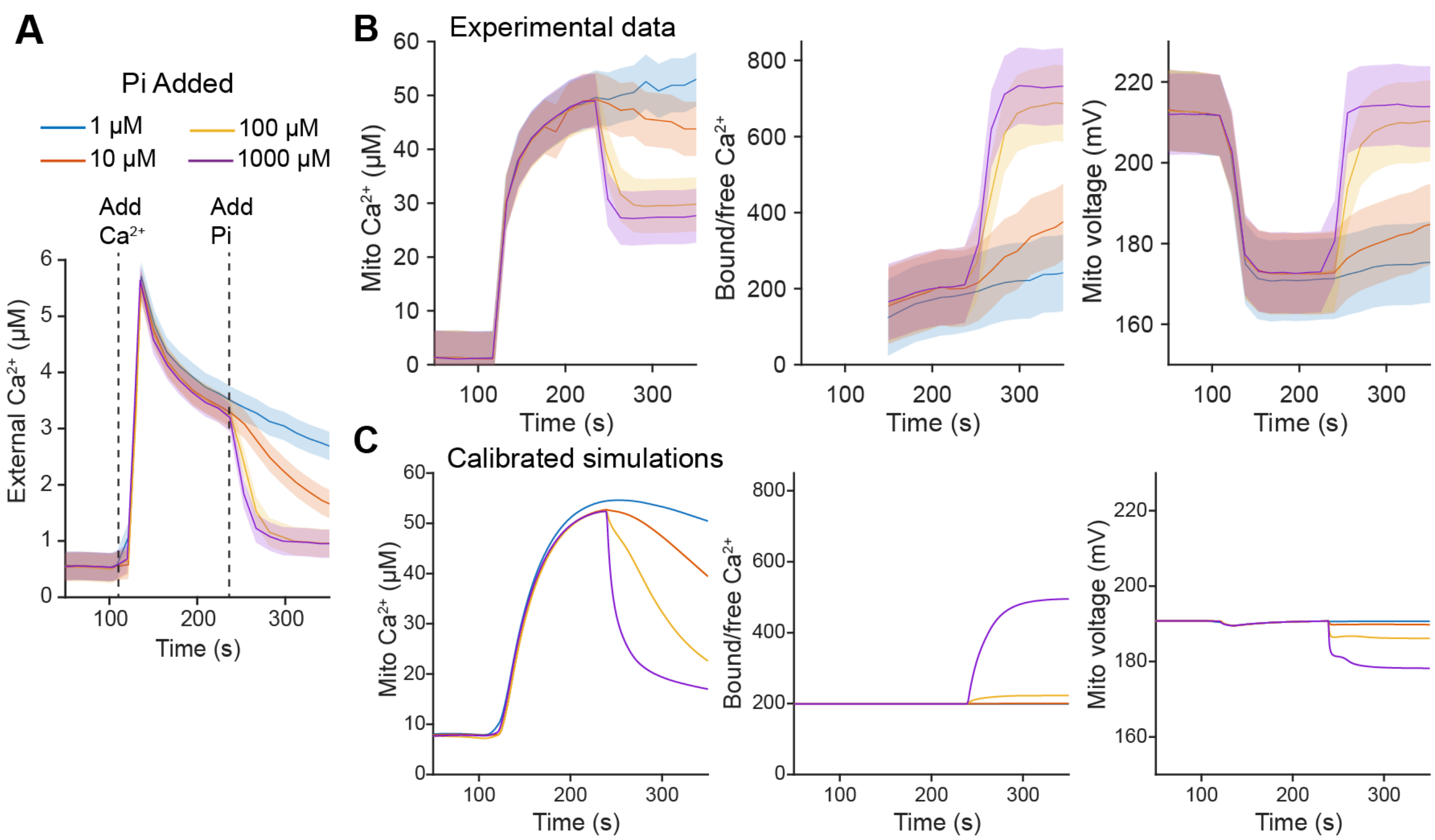

Figure A3: Calibration of mitochondrial Ca^{2+} and phosphate handling for Module 5. A) Experimental measurements of Ca^{2+} in the extra-mitochondrial solution from (Wei et al., 2015). The mean curve is used as a driving function in the simulations shown in panel C. B) Experimental measurements from (Wei et al., 2015) ($n = 3$ experiments in each case). Error bars in A and B indicate the assumed standard error of the mean in our calibration procedure (see Equation (17)). C) Predictions of our model with calibrated parameters as reported in Table A7, assuming time-dependent Ca^{2+} from panel A and time-dependent phosphate as a Heaviside function with the indicated concentration, starting at time $t = 240$ s. The minimized weighted sum of square errors (defined in Equation (17)) was 38.518.

bioRxiv preprint doi: https://doi.org/10.1101/2025.05.22.655415; this version posted July 24, 2026. The copyright holder for this preprint (which was not certified by peer review) is the author/funder, who has granted bioRxiv a license to display the preprint in perpetuity. It is made available under a CC-BY 4.0 International license.

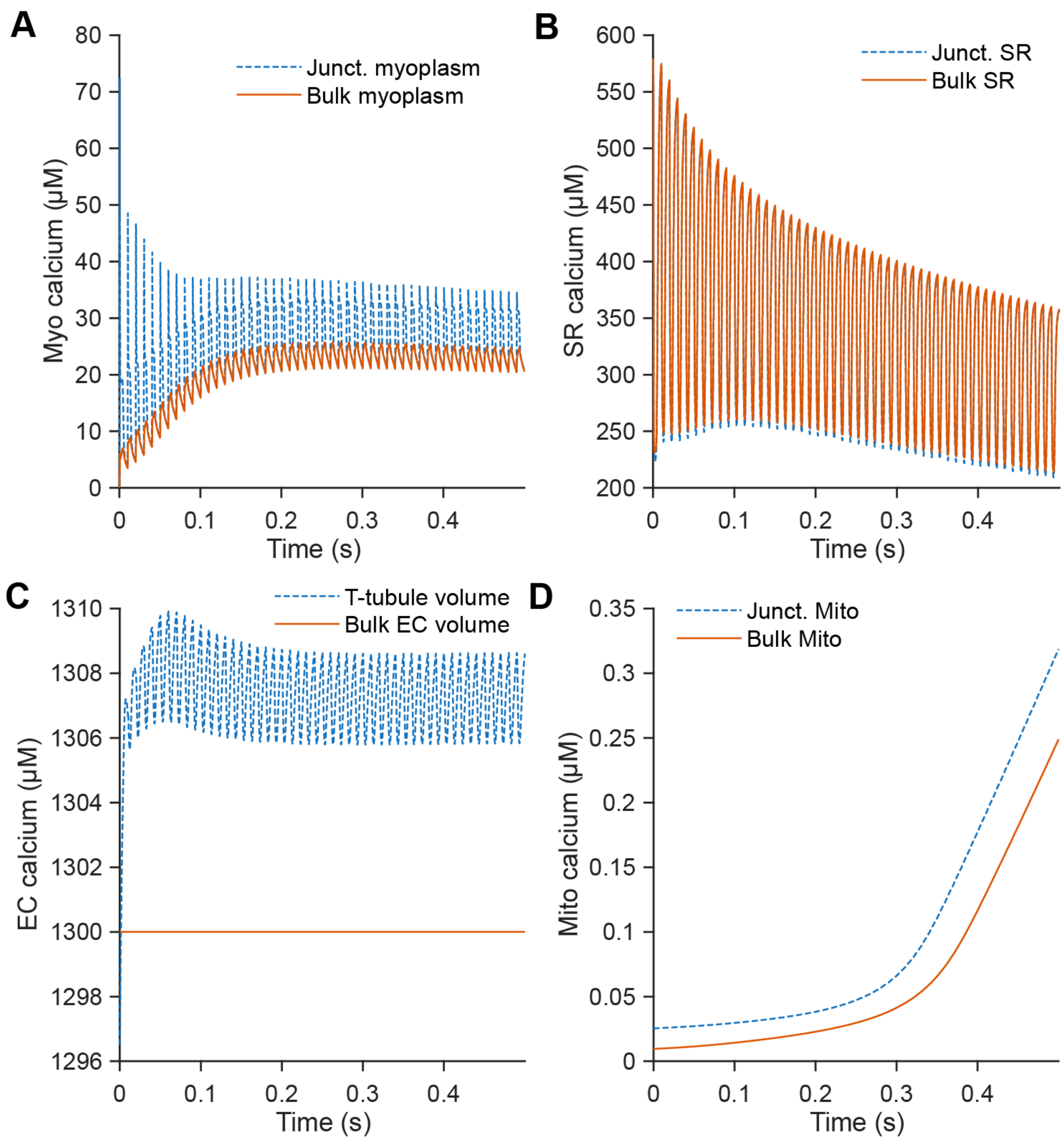

Figure A4: Calcium concentrations in different subregions of the compartmental model. A) Schematic summarizing the subregions of extracellular, myoplasmic, and SR compartments. B) Myoplasmic Ca^{2+} in the bulk vs. junctional myoplasm. B) SR Ca^{2+} in the bulk vs. junctional SR. C) Ca^{2+} in the bulk extracellular volume vs. the T-tubule volume. C) Ca^{2+} in bulk mitochondria vs. junctional mitochondria. All concentrations were computed from the same test conditions shown in Figure 3.

bioRxiv preprint doi: https://doi.org/10.1101/2025.05.22.655415; this version posted July 24, 2026. The copyright holder for this preprint (which was not certified by peer review) is the author/funder, who has granted bioRxiv a license to display the preprint in perpetuity. It is made available under a CC-BY 4.0 International license.

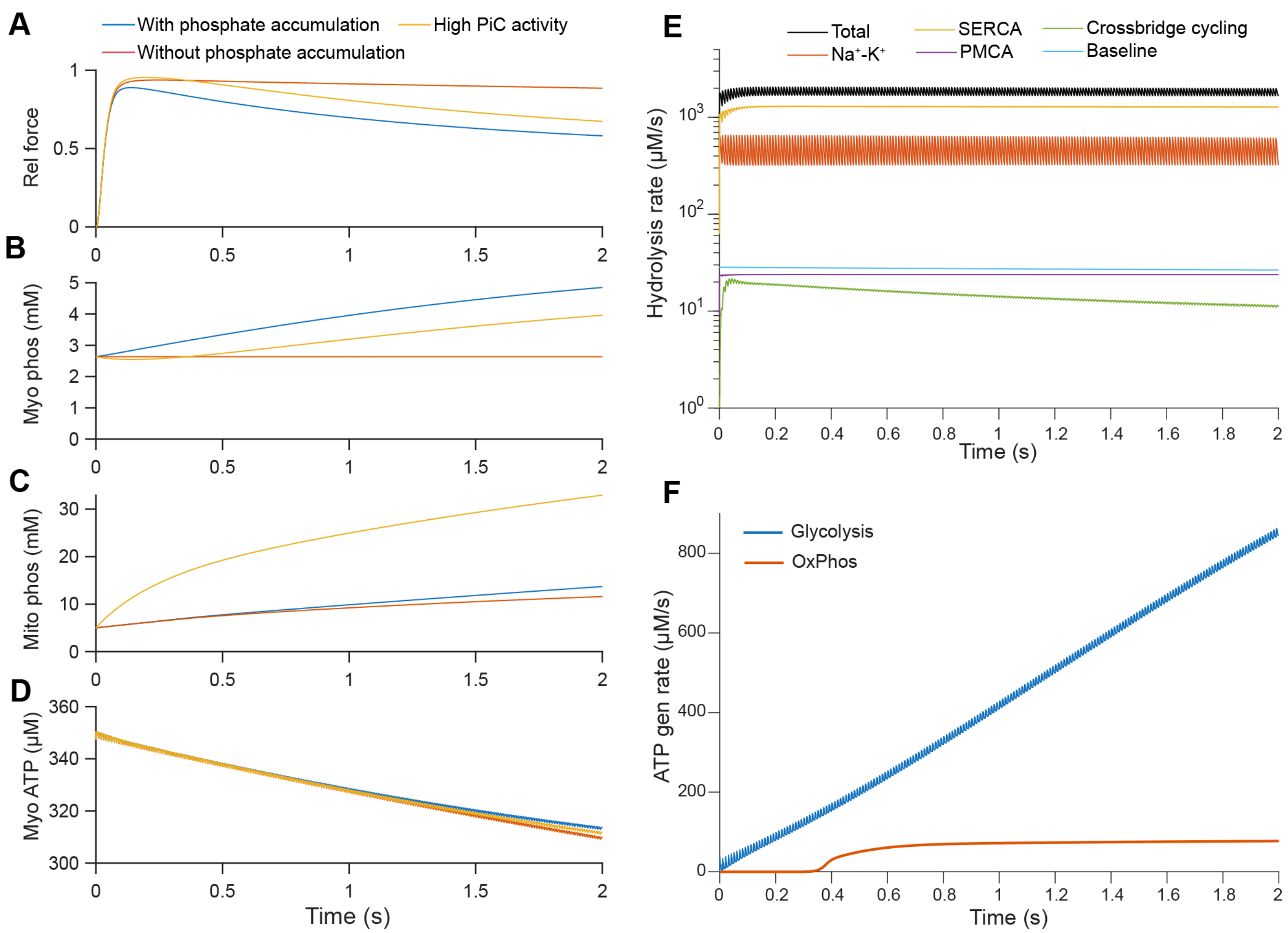

Figure A5: Effects of phosphate accumulation due to ATP hydrolysis in the myoplasm. A-D) Model predictions with and without phosphate accumulation, or with 10 times the baseline value for PiC permeability. Plots show relative force (A), myoplasmic phosphate (B), mitochondrial phosphate (C), and myoplasmic ATP (D). E) Contributions of each ATP-consuming process in the model for a simulation with normal phosphate accumulation. F) Contribution of glycolysis vs. oxidative phosphorylation to ATP generation over time for a simulation with normal phosphate accumulation.

bioRxiv preprint doi: https://doi.org/10.1101/2025.05.22.655415; this version posted July 24, 2026. The copyright holder for this preprint (which was not certified by peer review) is the author/funder, who has granted bioRxiv a license to display the preprint in perpetuity. It is made available under a CC-BY 4.0 International license.

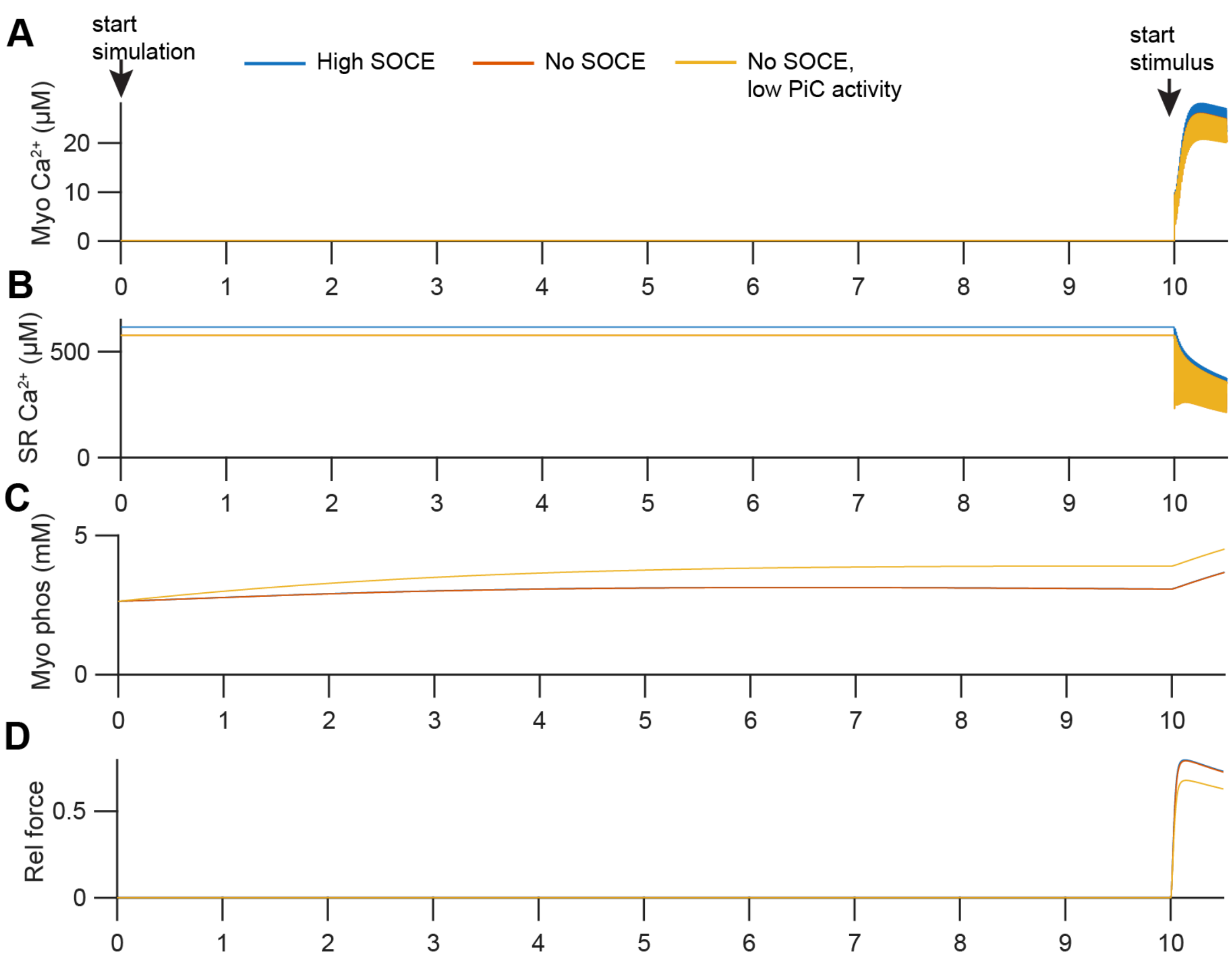

Figure A6: Initialization protocol for simulations shown in Figure 7. After normal initialization of the system (as described in Methods), these tests were run for 10 s before the stimulus occurred. As shown, myoplasmic (A) and SR (B) Ca^{2+} concentrations remain mainly constant prior to stimulus, whereas myoplasmic phosphate gradually accumulates due to resting ATP hydrolysis (C). The resulting predictions for relative force are shown in panel D.

bioRxiv preprint doi: https://doi.org/10.1101/2025.05.22.655415; this version posted July 24, 2026. The copyright holder for this preprint (which was not certified by peer review) is the author/funder, who has granted bioRxiv a license to display the preprint in perpetuity. It is made available under a CC-BY 4.0 International license.

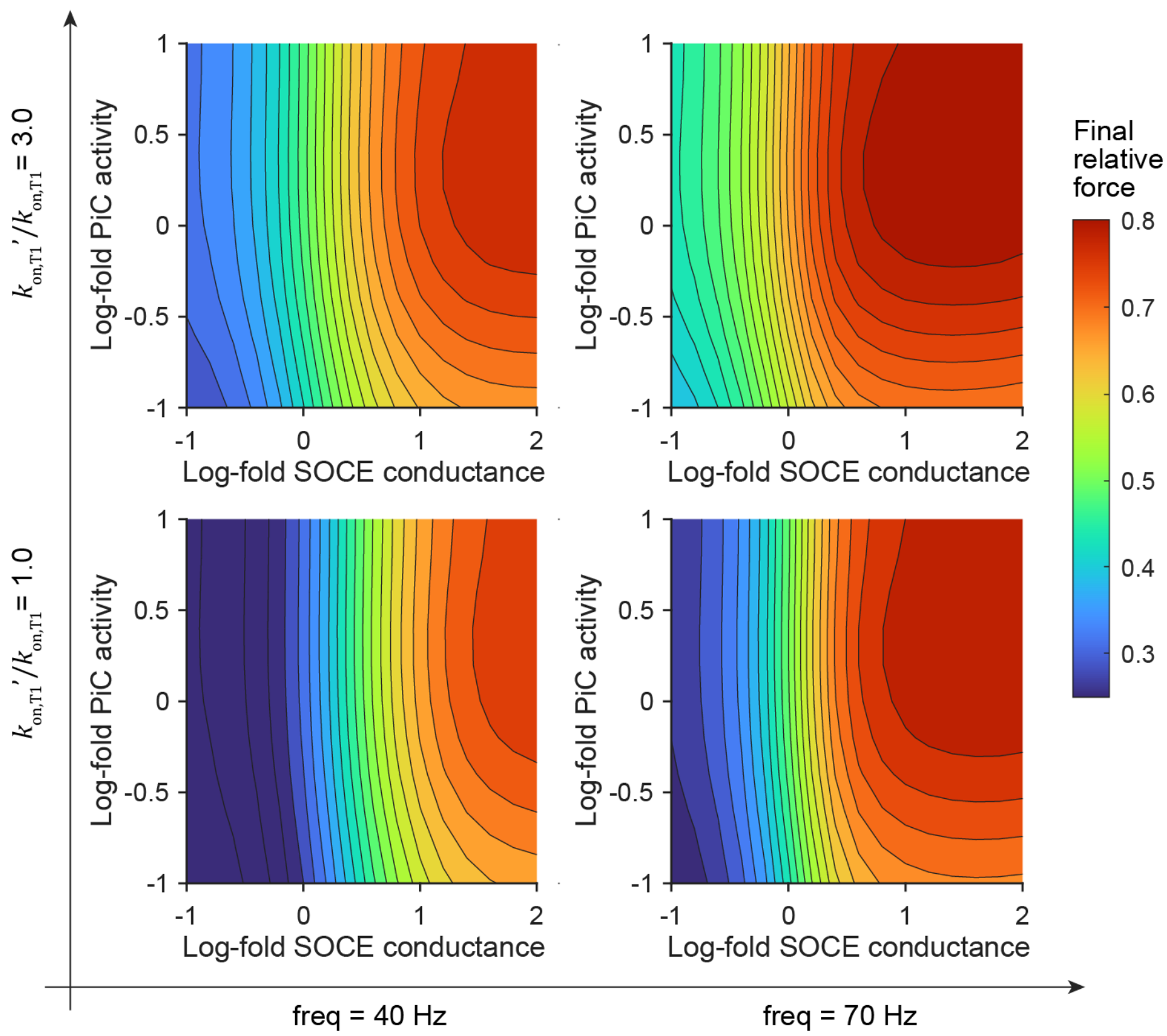

Figure A7: Effect of SOCE on force during resistance exercise, tested over different parameter regimes. Force as a function of Orai1 conductance and PiC activity in the final repetition of a 60 s resistance exercise simulation for stimulus frequencies of 40 Hz (left) or 70 Hz (right) and for troponin Ca^{2+} sensitivity at 100% (lower) or 300% (upper) of the estimated value.

bioRxiv preprint doi: https://doi.org/10.1101/2025.05.22.655415; this version posted July 24, 2026. The copyright holder for this preprint (which was not certified by peer review) is the author/funder, who has granted bioRxiv a license to display the preprint in perpetuity. It is made available under a CC-BY 4.0 International license.

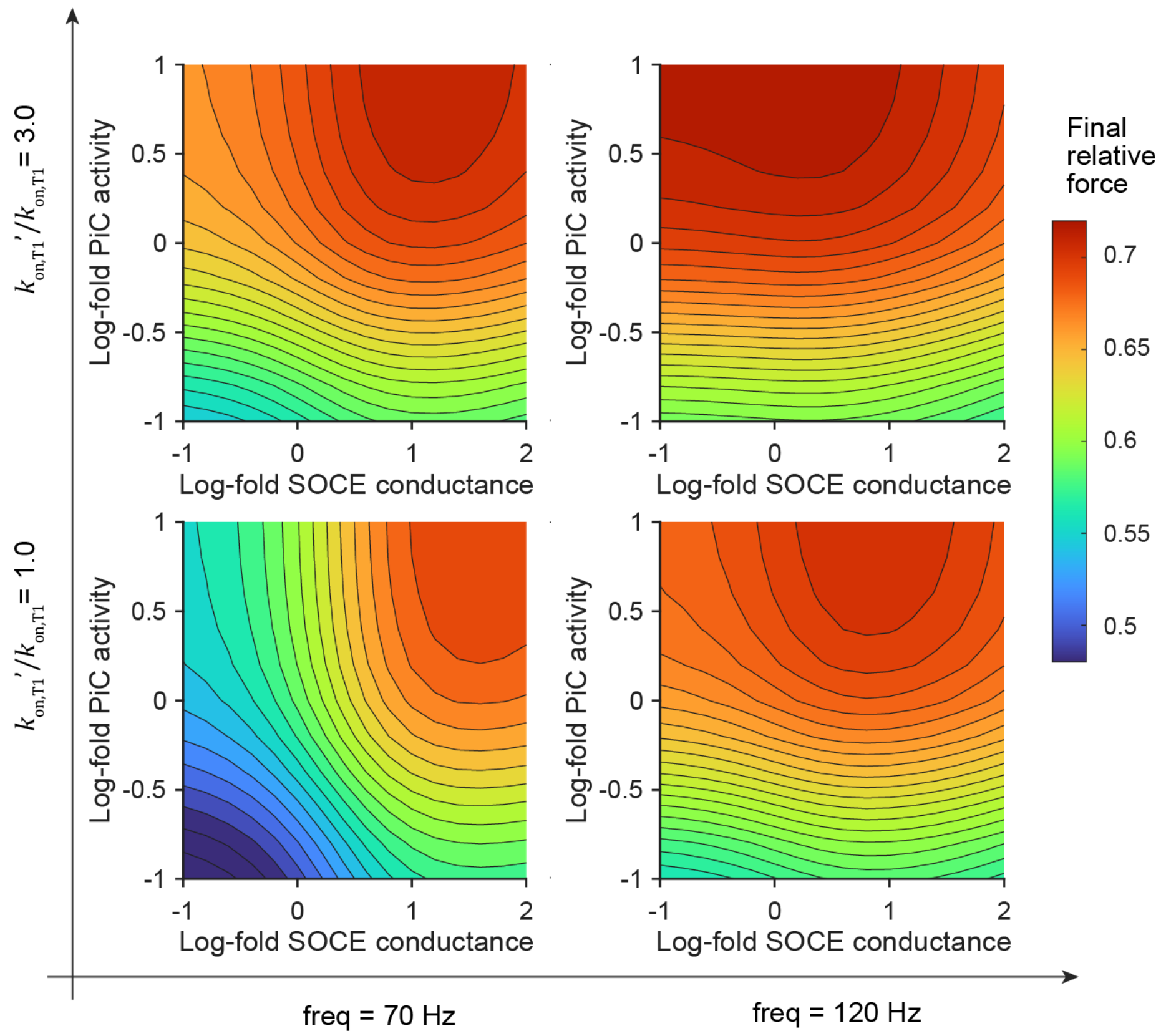

Figure A8: Effect of SOCE on force during high-intensity interval training (HIIT), tested over different parameter regimes. Force as a function of Orai1 conductance and PiC activity in the final stride of a 20 s HIIT simulation for stimulus frequencies of 70 Hz (left) or 120 Hz (right) and for troponin Ca^{2+} sensitivity at 100% (lower) or 300% (upper) of the estimated value.
